# mTORC1 promotes TOP mRNA translation through site-specific phosphorylation of LARP1

**DOI:** 10.1093/nar/gkaa1239

**Published:** 2021-01-04

**Authors:** Jian-Jun Jia, Roni M Lahr, Michael T Solgaard, Bruno J Moraes, Roberta Pointet, An-Dao Yang, Giovanna Celucci, Tyson E Graber, Huy-Dung Hoang, Marius R Niklaus, Izabella A Pena, Anne K Hollensen, Ewan M Smith, Malik Chaker-Margot, Leonie Anton, Christopher Dajadian, Mark Livingstone, Jaclyn Hearnden, Xu-Dong Wang, Yonghao Yu, Timm Maier, Christian K Damgaard, Andrea J Berman, Tommy Alain, Bruno D Fonseca

**Affiliations:** Children's Hospital of Eastern Ontario (CHEO) Research Institute, Department of Biochemistry, Microbiology and Immunology, University of Ottawa, Ottawa, Canada; Department of Biological Sciences, University of Pittsburgh, Pittsburgh, PA, USA; Department of Molecular Biology and Genetics, Aarhus University, Aarhus, Denmark; GABBA PhD Program, Abel Salazar Biomedical Sciences Institute, University of Porto, Porto, Portugal; PrimerGen Ltd, Viseu, Portugal; Children's Hospital of Eastern Ontario (CHEO) Research Institute, Department of Biochemistry, Microbiology and Immunology, University of Ottawa, Ottawa, Canada; Children's Hospital of Eastern Ontario (CHEO) Research Institute, Department of Biochemistry, Microbiology and Immunology, University of Ottawa, Ottawa, Canada; Children's Hospital of Eastern Ontario (CHEO) Research Institute, Department of Biochemistry, Microbiology and Immunology, University of Ottawa, Ottawa, Canada; Children's Hospital of Eastern Ontario (CHEO) Research Institute, Department of Biochemistry, Microbiology and Immunology, University of Ottawa, Ottawa, Canada; Children's Hospital of Eastern Ontario (CHEO) Research Institute, Department of Biochemistry, Microbiology and Immunology, University of Ottawa, Ottawa, Canada; Children's Hospital of Eastern Ontario (CHEO) Research Institute, Department of Biochemistry, Microbiology and Immunology, University of Ottawa, Ottawa, Canada; Children's Hospital of Eastern Ontario (CHEO) Research Institute, Department of Biochemistry, Microbiology and Immunology, University of Ottawa, Ottawa, Canada; Department of Molecular Biology and Genetics, Aarhus University, Aarhus, Denmark; Cancer Research UK Beatson Institute, Glasgow, UK; Biozentrum, University of Basel, Basel, Switzerland; Biozentrum, University of Basel, Basel, Switzerland; Department of Biochemistry, Rosalind and Morris Goodman Cancer Centre, McGill University, Montréal, Canada; Department of Biochemistry, Rosalind and Morris Goodman Cancer Centre, McGill University, Montréal, Canada; Department of Biochemistry, Rosalind and Morris Goodman Cancer Centre, McGill University, Montréal, Canada; Department of Biochemistry, UT Southwestern Medical Center, Dallas, TX, USA; Department of Biochemistry, UT Southwestern Medical Center, Dallas, TX, USA; Biozentrum, University of Basel, Basel, Switzerland; Department of Molecular Biology and Genetics, Aarhus University, Aarhus, Denmark; Department of Biological Sciences, University of Pittsburgh, Pittsburgh, PA, USA; Children's Hospital of Eastern Ontario (CHEO) Research Institute, Department of Biochemistry, Microbiology and Immunology, University of Ottawa, Ottawa, Canada; Children's Hospital of Eastern Ontario (CHEO) Research Institute, Department of Biochemistry, Microbiology and Immunology, University of Ottawa, Ottawa, Canada; PrimerGen Ltd, Viseu, Portugal

## Abstract

LARP1 is a key repressor of TOP mRNA translation. It binds the m^7^Gppp cap moiety and the adjacent 5′TOP motif of TOP mRNAs, thus impeding the assembly of the eIF4F complex on these transcripts. mTORC1 controls TOP mRNA translation *via* LARP1, but the details of the mechanism are unclear. Herein we elucidate the mechanism by which mTORC1 controls LARP1’s translation repression activity. We demonstrate that mTORC1 phosphorylates LARP1 *in vitro* and *in vivo*, activities that are efficiently inhibited by rapamycin and torin1. We uncover 26 rapamycin-sensitive phospho-serine and -threonine residues on LARP1 that are distributed in 7 clusters. Our data show that phosphorylation of a cluster of residues located proximally to the m^7^Gppp cap-binding DM15 region is particularly sensitive to rapamycin and regulates both the RNA-binding and the translation inhibitory activities of LARP1. Our results unravel a new model of translation control in which the La module (LaMod) and DM15 region of LARP1, both of which can directly interact with TOP mRNA, are differentially regulated: the LaMod remains constitutively bound to PABP (irrespective of the activation status of mTORC1), while the C-terminal DM15 ‘pendular hook’ engages the TOP mRNA 5′-end to repress translation, but only in conditions of mTORC1 inhibition.

## INTRODUCTION

mTORC1 plays a fundamental role in the control of mRNA translation (1–5), particularly for the class of transcripts known as TOP mRNAs, which bear a 5′terminal oligopyrimidine sequence immediately downstream of the N^7^-methyl guanosine triphosphate cap (6–8). The study of TOP mRNA translation is particularly interesting from a biochemical perspective because TOP mRNAs code for many of the protein components of the translation machinery, namely: all of the ribosomal proteins, many translation factors, in addition to some RNA-binding proteins (reviewed in (1,8)). Notably, while mTORC1 is known to regulate the translation other transcripts, TOP mRNAs comprise the class of transcripts that is most sensitive to translation repression by mTORC1 inhibitors (9–12). The translation of TOP mRNAs is exquisitely sensitive to mTORC1 inhibition achieved by pharmacological agents (13–15) and by deprivation of specific nutrients, including: amino acids (13–17), growth factors (18), hormones (19) and oxygen (20). Each of these nutrients activate distinct cellular signalling pathways; importantly, all of them converge on mTORC1. Chief among these pathways is the PI3K/ATK1/TSC/RHEB cascade that activates mTORC1 which, in turn, engages TOP mRNA translation (14,17,18,18–20). Interestingly, neither mTORC1 nor any of the components of the PI3K/ATK1/TSC/RHEB pathway physically interact with TOP transcripts. This observation led to the idea that an unidentified “missing protein or regulatory RNA component" physically interacted with both mTORC1 and TOP mRNAs thereby linking mTORC1 to the control of TOP mRNA translation (1,8). Compelling *in vitro* translation studies further suggested that this “missing link" likely functioned as a *repressor* (and not as an *activator*) of TOP mRNA translation (25). While the existence of a repressor of TOP mRNA translation has been recognized since 1999, the identity of said repressor and the precise mechanism by which mTORC1 controls its activity remained unclear until recently (8). The recent identification of LARP1 as a novel downstream target of mTORC1 (21,26) revealed the long-sought mechanism by which mTORC1 controls TOP mRNA translation (8).

The La-related protein 1 (LARP1) was recently identified by our group (21–23) (and subsequently confirmed by others (24)) as the elusive repressor of TOP mRNA translation downstream of mTORC1. Our data first revealed that: (i) La-related protein 1 (LARP1), an RNA-binding protein, is a translation repressor of TOP mRNAs that (ii) physically interacts with mTORC1, (iii) functions downstream of this complex and (iv) associates with TOP mRNAs in an mTORC1-dependent manner that is diametrically opposed to that of eIF4G1, whose (v) binding to TOP mRNAs is affected by altered levels of LARP1 in the cell (21). This led us to propose that mTORC1 controls TOP mRNA translation *via* LARP1 and that the latter represses TOP mRNA translation by competing with the eIF4F complex for binding to TOP mRNAs (21). The precise mechanism by which this occurs remained incompletely understood. Recent structural (23,27,28) and biochemical (24) data shed important new light into this process: LARP1 interacts with the m^7^Gppp cap and the adjacent 5′TOP motif *via* its conserved carboxy-terminal DM15 domain (23). In doing so, LARP1 effectively displaces eIF4E from the m^7^Gppp cap of TOP mRNAs and precludes the association of eIF4G1 with TOP mRNAs (21,23), thus blocking TOP mRNA translation (21,24).

How does mTORC1 dictate the inhibitory activity of LARP1? Typically, mTORC1 modulates the activity of its downstream targets through multisite phosphorylation of key serine and threonine residues. For instance, mTORC1 catalyzes the phosphorylation of multiple residues on ribosomal protein S6 kinases (S6Ks) (29–34), eukaryotic initiation factor 4E-binding proteins (4E-BPs) (35–46) and proline-rich AKT1 substrate 40kDa (PRAS40) (47–49), a less well-characterized substrate of mTORC1. 4E-BPs (of which there are three homologs in mammals: 4E-BP1, 4E-BP2 and 4E-BP3) and S6Ks (S6K1 and S6K2) are the most intensively studied direct mTORC1 substrates; accordingly, these targets are frequently referred to as the most important effectors of mTORC1 in mRNA translation (50). Two authoritative phosphoproteome studies (51,52) coupled the use of mTOR-specific pharmacological agents (rapamycin and torin1/Ku-0063794) to the power of liquid chromatography tandem mass spectrometry (LC-MS/MS) to reveal that, in addition to the well-characterized 4E-BPs and S6Ks, the mTORC1 pathway modulates the phosphorylation (either directly or indirectly by way of activation of downstream kinases) of thousands of presently uncharacterized mTORC1 substrates. LARP1 was identified as one such new mTORC1 substrate (51,52). mTORC1 directly catalyzes the phosphorylation of LARP1 *in vitro* (53,54), but the significance of this is presently unknown.

In this study, we demonstrate that mTORC1 catalyzes the phosphorylation of multiple serine and threonine residues in LARP1 both *in vitro* and *in vivo*. Specifically, we show that rapamycin alters the phosphorylation status of upwards of 26 serine and threonine LARP1 residues that are broadly distributed into 7 clusters. We observe that phosphorylation of different clusters has distinct functional consequences. For example, phosphorylation of key residues within clusters 4 and 5 (located in the mid-region of LARP1) enhances mTORC1 association with LARP1, while phosphorylation of key residues within cluster 6, which is proximal to the C-terminal DM15 region, impairs the RNA-binding and translation inhibitory activities of LARP1. Specifically, phosphorylation of cluster 6 residues hinders the binding of the DM15 region to the 5′UTR of RPS6 TOP mRNA. Consistent with this finding, we observe that phosphorylation of these residues abrogates the inhibitory effect of LARP1 on TOP mRNA translation. We observe herein that phosphorylation of cluster 6 is particularly sensitive to the mTORC1 inhibitor rapamycin. Rapamycin efficiently inhibits TOP mRNA translation (13–15) but, as shown here, does so only in the presence of a functional copy of the *LARP1* gene. Genetic CRISPR/Cas9 deletion of LARP1 renders TOP mRNA almost completely insensitive to rapamycin, indicating that mTORC1 promotes TOP mRNA translation primarily through inactivation of the LARP1 TOP mRNA translation repressor. We show that re-expression of the wildtype DM15 fragment of LARP1 restores TOP mRNA translation repression to LARP1^KO^ cells, while a phosphomimetic mutant bearing ten mutations for each of the phosphoresidues within cluster 6 fails to do so. Collectively, these findings provide the first evidence for a functional regulatory role for mTORC1-mediated LARP1 phosphorylation on TOP mRNA binding and translation de-repression. Further, we present a refined version of our original repression model, herein referred to as the ‘pendular hook’ repression model.

## MATERIALS AND METHODS

### Mammalian cell culture, transfection and lysis

HEK 293T cells were used in every experiment shown herein. Cells were cultured/treated in 10-cm tissue culture-treated polystyrene dishes (Corning, catalogue no. 430167) at 37°C in a humidified incubator at 5% (v/v) CO_2_. Dulbecco's modified Eagle's media (DMEM) High Glucose (HyClone GE Healthcare, catalogue no. SH30022.01) supplemented with 10% (v/v) fetal bovine serum (Millipore Sigma, catalogue no. F1051) and 100 units/ml penicillin/streptomycin (HyClone GE Healthcare, catalogue no. SV30010)—designated here for ease as complete growth media—was used for cell propagation and treatments. For experiments requiring activation of mTORC1 cells were propagated to near-confluency (∼80%) in complete growth media, at which point the media was aspirated and replenished with fresh complete growth media for 3 h. Where indicated cells were simultaneously treated (3 h) with 100 nM rapamycin (LC laboratories, catalogue no. R-5000), 300 nM torin1 (Tocris, catalogue no. 4247), 10 μM PF-4708671 (Tocris, catalogue no. 4032), 10 μM MK-2206 (Cayman Chemicals, catalogue no. 11593) or 30 μM LY294002 (LC laboratories, catalogue #L-7962) or 0.1% (v/v) dimethyl sulfoxide (DMSO) (Millipore Sigma, catalogue no. D1435). DMSO was used as the solvent in the resuspension of every chemical listed above. Where indicated cells were transiently transfected with plasmid DNA for mammalian expression using lipofectamine 2000 reagent (Invitrogen by Thermo Fisher Scientific, catalogue no. 11668-019) as per manufacturer's instructions. Typically, 4–8 μg of plasmid DNA were used to transfect a 10-cm petri dish of near-confluent HEK 293T cells. Cells were transfected by incubating the plasmid DNA/lipofectamine 2000 mix in Opti-MEM I (Invitrogen by Thermo Fischer Scientific, catalogue no. 22600-050) for 3–4 h at 37°C in a 5% (v/v) CO_2_ humidified incubator. Transfected cells were then incubated in complete growth media for 24 h followed by another media change for 3 h in complete growth media to activate mTORC1. After mTORC1 stimulation by media change, cells were washed in 5 ml sterile ice-cold phosphate buffered saline (PBS) (important to incline the plate and aspirate all the PBS such that it does not dilute out the lysis buffer) and subsequently lysed in 1 ml of extraction buffer (40 mM HEPES (pH 7.5, room temperature), 0.3% (w/v) CHAPS zwitterionic detergent, 120 mM NaCl, 1mM EDTA, 10 mM sodium pyrophosphate, 10 mM β-glycerophosphate, 50 mM sodium fluoride, 1.5 mM sodium orthovanadate, 1 mM dithiothreitol (DTT) and cOmplete™ Mini EDTA-free protease inhibitor cocktail tablets (Millipore Sigma, catalogue no. 04693159001) and 1–100 μg/ml RNase A (Millipore Sigma, catalogue no. 10109169001) for 1 h at 4°C. RNase A was reconstituted in 10 mM Tris–HCl (pH 7.5), 15 mM NaCl and 50% (v/v) glycerol to a final concentration of 10 mg/ml and heated at 96°C for 15 min to inactivate contaminating DNases, then cooled down slowly to room temperature. A range of RNase A concentrations (0.4–4 μg/ml) can be used in the extraction buffer to digest RNA and enhance the interaction between endogenous LARP1 and RAPTOR proteins (Figure 5A). A final concentration of at least 0.4 μg/ml RNase A in the extraction buffer is recommended for optimal interaction of RAPTOR with LARP1. RNase A was omitted from the extraction buffer for RNA immunoprecipitation experiments. CHAPS detergent is considerably weaker than most other detergents and, as such, cells must be incubated with extraction buffer for at least 1h before scraping for efficient lysis. Cells were scraped and lysates pre-cleared by centrifugation at 16 000 × *g* for 10 min at 4°C. Supernatant was collected onto a fresh microfuge tube. Lysate samples destined for SDS-PAGE.western blot analysis were prepared by adding 50 μl of 4× sample buffer to 150 μl of lysate.

### RNA- and protein-immunoprecipitation

HEK 293T cell lysates were prepared as described above. One important difference between RNA- and protein-IPs is that RNase A was omitted from the extraction buffer for RNA immunoprecipitation but not for protein immunoprecipitation. In addition, 750 μl of lysate was used for RNA immunoprecipitation and 500 μl of lysate was used for protein immunoprecipitation. RNA and protein immunoprecipitation were carried out as follows: 5 μl of LARP1 antibody (AbCam, catalog no. 86359) were added to lysates and incubated for 1 h 30 min rotating end-over-end at 4°C. Then added 35 μl of pre-washed protein G-conjugated magnetic Dynabeads (Life Technologies by Thermo Fisher Scientific, catalogue no. 10003D) were added to the antibody/lysate mixture and incubated for 1 h rotating end-over-end at 4°C. Following the 1 h incubation, the beads were pelleted by centrifugation at 1000 × *g* for 5 min on a tabletop centrifuge at 4°C, the supernatant aspirated and collected for analysis of unbound material. The beads were then washed twice with 1 ml of extraction buffer. After washing, 500 μl of Trizol reagent (Life Technologies by Thermo Fisher Scientific, catalog no. 15596018) was added—followed by vortexing for 5 s. We then added an identical volume (500 μl ) of extraction buffer to the mix, such that the volume of Trizol to aqueous phase was 1:1 (adding Trizol reagent to the beads prior to adding extraction buffer allows for maximal recovery of RNA from beads) and vortexed samples for an additional 5 s. Samples were stored overnight at –80°C.

### RNA extraction, cDNA synthesis and reverse transcription-digital droplet PCR (RT-ddPCR)

Samples were thawed from –80°C at room temperature, 50 μl of chloroform was added and then vortexed for 15 s followed by incubation for 15 min at room temperature. Then subjected to centrifugation at 21 000 × *g* for 15 min at 4°C. The aqueous phase was collected, and an equal volume of 100% isopropanol was added (to precipitate total RNA) and samples vortexed for 15 s. Samples were finally incubated at –20°C to enhance precipitation. We next thawed the samples from –20°C and centrifuged them at 21 000 × *g* for 15 min at 4°C. Supernatants were subsequently discarded, and pellet washed gently with 1 ml 75% (v/v) ice-cold ethanol. The centrifugation step was repeated (21 000 × *g* for 5 min at 4°C) and the supernatants discarded. RNA pellets were air-dried overnight at room temperature and suspended in 100 μl RNase-free water (Millipore Sigma, catalogue no. W4502-1L) for inputs and 10 μl for immunoprecipitates. The reverse-transcription reaction was carried out using the iScript Select cDNA synthesis kit (BioRad, catalogue no. 170-8897) as per manufacturer's protocol with modifications. Briefly, 4 μl of 5× Select reaction mix were added to 1 μl iScript reverse transcriptase, 2 μl Oligo(dT)20 and 10 μl RNA supplemented with RNase-free water (Millipore Sigma, catalogue no. W4502-1L) to a final volume of 20 μl. The reaction mix was incubated at 42°C for 1 h, followed by 85°C for 5 min. The cDNA reaction product was then diluted 500× in RNase-free water (Millipore Sigma, catalogue no. W4502-1L) prior to analysis by digital droplet PCR (ddPCR). Each ddPCR reaction was carried by adding 10 μl QX200™ ddPCR EvaGreen Supermix (BioRad, catalogue no. 186-4034), 0.2 μl of each primer at a stock concentration of 10 μM, 8 μl of diluted cDNA and 1.6 μl RNase-free water (Millipore Sigma, catalogue no. W4502–1L) to a final reaction volume of 20 μl. The reaction mixtures were transferred to DG8™ Cartridges for QX100™/QX200™ Droplet Generator (BioRad, catalogue no. 186-4008) and 70 μl Droplet Generation Oil for EvaGreen (BioRad, catalogue no. 186-4006). Samples were emulsified on the Droplet Generator and subsequently transferred to a 96-well ddPCR plate. The plate was sealed with aluminum foil and the thermal cycling step ran using the following conditions: 95°C for 5 min, 95°C for 30 s, ramp down 2°C/s till it reached 62°C, then ramp up to 95°C and repeat this cycle 45 times. Lastly, samples were cooled down to 4°C for 5 min, heated up to 95°C for 5 min and held at 12°C indefinitely. Samples were analyzed on Biorad QX200™ Droplet Plate Reader.

### Sucrose gradient ultracentrifugation—polysome profiling analysis

Sucrose gradients (10–50%) were prepared in 20 mM HEPES/KOH (pH 7.6) at room temperature also containing 100 mM KCl, 5 mM MgCl_2_, 100 μg/ml cycloheximide (prepared fresh), EDTA-free protease inhibitors cocktail tablets (catalogue no. 04693 132 001, Roche Applied Sciences) and 200 units/ml RNasin^®^ ribonuclease inhibitor (catalogue no. N2515, Promega). Isolation of polysomes was carried out as follows: HEK 293T cells were cultured to sub-confluency (∼70%) (in 15 cm tissue-culture treated dishes) at which point cells were re-fed by replacing exhausted media with fresh complete growth media (DMEM supplemented with 10% (v/v) fetal bovine serum, 100 units/ml penicillin G, and 100 μg/ml streptomycin sulfate at 37°C and 5% (v/v) CO_2_ humidified incubator) for 3 h in the presence of 0.1% (v/v) DMSO (vehicle), 100 nM rapamycin or 300 nM torin1. In the last 5 min of treatment, freshly prepared cycloheximide was added to the cells to a final concentration of 100 μg/ml. Treatment with cycloheximide locks the ribosome onto the mRNA thus minimizing ribosome run-off. Cells were washed once in ice-cold phosphate buffered saline (PBS) supplemented with 100 μg/ml cycloheximide and then scraped gently with a cell scraper in 14 ml of ice-cold PBS containing 100 μg/ml cycloheximide. Cells were centrifuged at 200 × *g* for 5 min at 4°C on a tabletop centrifuge and resuspended in 900 μl of hypotonic buffer composed of 5 mM Tris (pH 7.5 at room temperature), 2.5 mM MgCl_2_, 1.5 mM KCl, EDTA-free protease inhibitors cocktail tablets (catalogue no. 04693 132 001, Roche Applied Science), 100 μg/ml RNasin, 0.5% (v/v) Triton X-100 and 0.5% (w/v) sodium deoxycholate) and vortexed for 5 s. Lysates were pre-cleared by centrifugation at 21 000 × *g* for 5 min at 4°C. Supernatant was collected and transferred onto a fresh microfuge tube. Absorbance at 260 nm (*A*_260 nm_) was determined for each sample. Five hundred micrograms of total RNA were typically loaded onto each gradient. Gradients were subjected to ultracentrifugation at 36 000 × *g* for 2 h at 4°C on a SW41Ti rotor in a Beckman Coulter (Optima L80 XP) ultracentrifuge. Brake was set at 5. Centrifuged samples were subjected to fractionation into 14 fractions (750 μl each) using a Teledyne ISCO fractionation system. Absorbance 254 nm (*A*_254 nm_) was monitored with an UV–visible detector (Brandel). Data were analyzed with WinDAQ software. To each fraction was added an equal volume of Trizol, vortexed for 30 s and frozen at –80°C until further analysis. Total RNA was extracted from each fraction as detailed above.

### Sodium dodecyl sulfate polyacrylamide gel electrophoresis (SDS-PAGE) and western blot

Total protein levels and phosphorylation were monitored by SDS-PAGE/western blot. Lysates were resolved in 10% (w/v) acrylamide (Millipore Sigma, catalogue no. A3553-500G) gels (1.5 mm thickness) containing 0.1% (w/v) bis *N*,*N*′-methylene bisacrylamide (BioRad, catalogue no. 161-0201) at a ratio of 100:1 of acrylamide to bis *N*,*N*′-methylene bisacrylamide. Proteins were then transferred onto an 0.2 μm nitrocellulose membrane (BioRad, catalogue no. 1620112) for 1 h 30 min at constant 100 V by wet (immersion) transfer in a modified Towbin 1× transfer buffer (25 mM Tris, 192 mM glycine at pH 8.3) (55) containing low (10% (v/v)) methanol and 0.1% (w/v) sodium dodecyl sulfate (SDS) for easier transfer of larger molecular weight proteins. The membrane was then blocked in 5% (w/v) non-fat milk suspended in Tris buffer saline (1× TBS, 150 mM NaCl, 2.7 mM KCl and 24.7 mM Tris base at pH 7.6) containing 0.02% (v/v) Tween20 (TBS-T) for 1 h at room temperature followed by incubation with primary antibodies in 5% bovine serum albumin (BSA heat shock fraction) (Millipore Sigma, catalogue no. A7906) in TBS-T overnight at 4°C on an orbital shaker. Membranes were then washed twice for 5 min each time in TBS-T and incubated with HRP-conjugated secondary antibodies for 45 min at room temperature on an orbital shaker. Unbound antibody was washed by rinsing membranes thrice in TBS-T for 5 min each time. Protein was detected by enhanced chemiluminescence (ECL) using the western lightning plus-ECL reagent (Perkin Elmer Inc., catalogue no. NEL105001EA). ECL signal was detected by autoradiography using HyBlot CL film (Denville Scientific Inc., catalogue no. E3018). All proteins were analyzed as aforementioned with exception to 4E-BPs; the latter were resolved on 13.5% (w/v) acrylamide gels (1.5 mm thickness) containing 0.36% (w/v) bis *N*,*N*′-methylene bisacrylamide (BioRad, catalogue no. 161-0201) at a ratio of 37.5:1 of acrylamide to bis-*N*,*N*′-methylene bisacrylamide). Proteins were then transferred onto an 0.2 μm nitrocellulose membrane (BioRad, catalogue no. 1620112) for 1h at constant 100 V by wet (immersion) transfer in a modified Towbin 1× transfer buffer (25 mM Tris, 192 mM glycine at pH 8.3) (55) containing high (20% (v/v)) methanol and 0.1% (w/v) sodium dodecyl sulfate (SDS). Higher methanol percentage is used to avoid over-transfer of low molecular weight proteins (such as 4E-BP1). To enhance the retention of 4E-BP1 protein on the membrane, the proteins were crosslinked to the membrane by incubating the membrane for 30 min with 0.05% (v/v) glutaraldehyde solution (Bio Basic Canada Inc., catalogue no. GC3870) prepared in phosphate buffered saline (1× PBS: 137 mM NaCl, 2.7 mM KCl, 10 mM Na_2_HPO_4,_ 2 mM KH_2_PO_4_ at pH 7.4) on an orbital shaker. Membranes were rinsed twice with deionized water and subsequently blocked with non-fat milk, probed with primary/secondary antibodies and developed as described above.

### Antibody sources

Anti-human LARP1 rabbit polyclonal antibody (catalogue no. ab86359) and anti-human PABP rabbit polyclonal antibody (catalogue no. ab21060) were purchased from Abcam. Anti-FLAG M2 mouse monoclonal antibody (catalogue no. F1804) and anti-rabbit horseradish peroxidase (HRP)-conjugated IgG (catalogue no. A0545) were purchased from Millipore Sigma. Anti-human RAPTOR rabbit polyclonal antibody (catalogue no. A300-553A) was purchased from Bethyl Laboratories. Anti-human RPS6 (C-8) mouse monoclonal IgG antibody (catalogue no. sc-74459) and anti-human S6K1 (C-18) rabbit polyclonal IgG antibody were purchased from Santa Cruz Biotechnology. Anti-rabbit eIF4E1 mouse IgG antibody (catalogue no. 610269) was purchased from BD Transduction Laboratories. Anti-human mTOR rabbit monoclonal (7C10) antibody (catalogue no. 2983), anti-phospho S473 human AKT1 rabbit polyclonal antibody (catalogue no. 9271S), anti-human AKT1 pan rabbit monoclonal (C67E7) antibody (catalogue no. 4691S), anti-hemagglutinin (HA) rabbit monoclonal (C29F4) antibody (catalogue no. 3724), anti-human eIF4G1 rabbit monoclonal (C45A4) antibody (catalogue no. 2469S), anti-phospho T389 human S6K1 rabbit monoclonal (108D2) antibody (catalogue no. 9234), anti-human S6K1, anti-phospho T37/T46 human 4E-BP1 rabbit monoclonal (236B4) antibody (catalogue no. 2855), anti-phospho S240/S244 human RPS6 rabbit polyclonal antibody (catalogue no. 2215S), anti-human 4E-BP1 rabbit monoclonal (53H11) antibody (catalogue no. 9644S) and anti-mouse horseradish peroxidase (HRP)-conjugated IgG (catalogue no. 7076S) antibody were purchased from Cell Signalling Technology. Primary antibodies were used at 1:1000 dilution, while secondary antibodies were used at 1:10 000.

### Generation of plasmid DNAs

pCMV6-entry human wildtype LARP1 transcript variant 1 (accession number NM_0153315) myc/FLAG-tagged (originally described in (21)) (catalogue no. RC200935) was purchased from Origene. pCMV6-human wildtype LARP2 transcript variant 1 (accession number NM_018078) (catalogue no. RC213675) and transcript variant 3 (accession no. NM_032239) (catalogue no. RC219586) myc/FLAG-tagged were also purchased from Origene. pCMV2 FLAG-tagged human wildtype La (56), pCMV2 FLAG-tagged human wildtype LARP4 (56), pCMV2 FLAG-tagged human wildtype LARP5 (56), human wildtype LARP6 (56) and human wildtype LARP7 were kindly gifted to us by Dr Richard J. Maraia (NIHCHD, Bethesda, MD, USA). pCMV6-entry human LARP1 R840E/Y883A double mutant has been previously generated and described (23).

### Generation of LARP1 CRISPR/Cas9 knockout (LARP1^KO^) HEK 293T cell lines

To create plasmids for expression of LARP1-specific gRNAs, sense (5′-CACCGAGACACATACCTGCCAATCG-3′) and antisense (5′-AAACCGATTGGCAGGTATGTGTCTC-3′) oligonucleotides were annealed and cloned into Esp3I-digested LentiCRISPRv2, resulting in a vector designated LentiCRISPR-LARP1gRNA. HEK 293T cells were maintained in DMEM medium (Gibco/Invitrogen by Thermo Fisher Scientific) supplemented with 10% fetal calf serum (Gibco/Invitrogen by Thermo Fisher Scientific) and 100 units/ml penicillin/streptomycin (Gibco/Invitrogen by Thermo Fisher Scientific, catalogue no. 15140122). All cells were cultured at 37°C in 5% (v/v) CO_2_. One day prior to transfection, HEK 293T cells were seeded at a density of 3 × 10^5^ cells/well in six-well plates. Transfections were carried out using 1 μg LentiCRISPR-LARP1gRNA and Lipofectamine 2000 Transfection Reagent (Invitrogen by Thermo Fisher Scientific, catalogue no. 11668-019) according to the manufacturer's protocol. Two days after transfection, the cells were reseeded at a density of 0.2 cells/well in 96-well plates. After expansion of single cells, genomic DNA was purified using the GenElute Mammalian Genomic DNA Miniprep Kits (Millipore Sigma-Aldrich, catalogue no. G1N10) according to the manufacturer's protocol. CRISPR/Cas9 LARP1^KO^ was verified by PCR on genomic DNA using the primers 5′-GGGAAAGGGATCTGCCCAAG-3′ and 5′-CACCAGCCCCATCACTCTTC-3′ and a Pfu Ultra II DNA polymerase (Agilent Technologies) according to the manufacturer's protocol followed by Sanger sequencing of the resulting PCR-product (GATC Biotech) using the primer 5′-GGGAAAGGGATCTGCCCAAG-3′.

### Site-directed mutagenesis and oligonucleotides

Phosphorylation and RNA-binding mutants of human LARP1 were generated by site-directed mutagenesis using Pfu Ultra HF DNA polymerase (Agilent Technologies, catalogue no. 600380-51) as described in the manufacturer's protocol. Oligonucleotides were designed using the PrimerGenesis software tool for automated oligonucleotide design (http://molecular-biology.primergen.group/). See Supplemental Table S1 for list of oligonucleotides employed on site-directed mutagenesis and sequence of human LARP1. See Supplemental Table S2 for list of oligonucleotides used to sequence human LARP1. See Supplemental Table S3 for list of oligonucleotides used on the analysis of human RPS6, RPL32, LDHA and β-actin mRNA levels by RT-ddPCR.

### Isoelectric focusing

Briefly, isoelectric focusing was performed as described in the manufacturer's manual. In detail, HEK 293T cells were stimulated with complete growth media (as described above) for 3h in the presence of 0.1% (v/v) DMSO, 100 nM rapamycin or 300 nM torin1. *Lysis:* Cells were then lysed by incubating in 800 μl rehydration buffer for 1 h at 4°C at which point cells were scraped, lysates pre-cleared by centrifugation at 16 000 × *g* for 10 min at 4°C. *Sample cleanup:* Samples were then further cleaned by using the ReadyPrep™ 2D Cleanup Kit: 200 μl of lysate were transferred into a clean microfuge tube, 600 μl precipitation agent 1 were added to the lysate and the microfuge tube vortexed. Vortexed sample was incubated for 15 min on ice. 600 μl of precipitation agent 2 were then added to the mixture of lysate and precipitation agent 1 and the microfuge tube vortexed. Samples were then centrifuged at 21 000 × *g* for 5 min to form a light pellet. Supernatant was discarded without disturbing the pellet. Residual liquid in the microfuge tube was collected and discarded following centrifugation for 30 s at 21 000 × *g*. 40 μl of wash reagent 1 was added on top of the pellet, the tube was vortexed and centrifuged 21 000 × *g* for 5 min. The wash was then discarded with a pipette and 25 μl of ReadyPrep proteomic grade water (BioRad, catalogue no. 163-2091). Tube was vortexed for 20 s. 1 ml of wash reagent 2 (pre-chilled at –20°C) and 5 μl of wash 2 additive was added to the tube. The tube was vortexed for 1 min. Samples in the tube were then incubated at –20°C for 30 min. Samples were vortexed once for 30 s midway through the incubation. After the incubation period, samples were centrifuged at 21 000 × *g* for 5 min to form a tight pellet. The supernatant was discarded, the tube centrifuged briefly for 30 s to discard any remaining wash. The pellet was air-dried at room temperature for ∼5 min until it looked translucent. The pellet was then resuspended in 2D rehydration buffer (see preparation below), vortexed for ∼3 min (or until the pellet was fully resuspended). Sample was centrifuged at 21 000 × *g* for 5 min at room temperature to clarify the protein sample and the supernatant used for IEF in IPG strips. *Sample application to IPG strip:* the IPG strip (pH 7–10, 11 cm) was thawed from –20°C and the rehydration/sample buffer lyophilized powder reconstituted by adding 6.1 ml of nanopure water supplied with the kit. We applied 185 μl of sample along the back edge of the channel of the rehydration/equilibration tray evenly, leaving 1 cm at each end. This step was repeated for each sample using a different channel. Once the protein samples were loaded into the rehydration tray, forceps were used to peel the coversheet from the thawed ReadyStrip IPG strip and the strip was placed over the sample in the rehydration/equilibration tray with the gel side facing down onto the sample. Air bubbles trapped underneath the strip were carefully removed by gently lifting the strip up and down with the forceps, or as a last resort, gently pressing down the strip. Each strip was then overlaid with 2 ml of mineral oil (BioRad, catalogue no. 163-2129) to prevent evaporation during the rehydration process. Mineral oil was added slowly to the plastic backing of the strips while moving the pipet along the length of the strip to avoid mineral oil seeping beneath the strip. The rehydration/equilibration tray was covered with the plastic lid provided and the tray left sitting on a level bench overnight (11–16 h) at room temperature to rehydrate the IPG strips and load the protein sample. *Isoelectric focusing:* Using forceps, paper wicks were placed at both ends of the Protean IEF focusing tray covering the wire electrodes. 8 μl of nanopure water (provided with kit) were added onto each wick. IPG strips were picked up from the rehydration/equilibration tray using forceps and held vertically for 10 s over filter paper to allow the mineral oil to drain. Draining the oil is important to remove the unabsorbed protein which would otherwise cause horizontal streaking. IPG strips were then transferred to the corresponding channel in the Protean IEF tray with the gel side facing down. Once placed in the focusing tray the strips were again covered with 2 ml of fresh mineral oil. Any trapped air bubbles were removed as described above. The focusing tray was covered with the lid and placed on the Protean IEF cell and the cover closed. Strips were resolved using the following 3-step protocol. Step 1: 250 V for 20 min and linear ramp; Step 2: 8000 V for 2 h 30 min and linear ramp; Step 3: 8000 V for an undefined time until it reached 20 000 V h with rapid ramping. This protocol takes an approximate time of 6 h and accumulated voltage of ∼30 000 V. A default cell temperature of 20°C with a maximum current of 50 μA/strip and no rehydration parameters were used. *Equilibration of IPG strips*: following completion of the electrophoresis step, the mineral oil was drained from IPG strips by holding them vertically using forceps for 10 s over filter paper and the strips were then transferred onto a clean/dry disposable rehydration/equilibration tray with the gel side facing up. Equilibration buffers 1 (containing 6 M urea, 2% (w/v) sodium dodecyl sulfate in 50 mM Tris–HCl buffer, pH 8.8 and 10 mg/ml dithiothreitol) was prepared by adding 13.35 ml of 30% (v/v) glycerol solution, supplied in the kit, to the buffer. Equilibration buffer 2 (containing 6 M urea, 2% (w/v) sodium dodecyl sulfate in 50 mM Tris–HCl buffer, pH 8.8) was prepared by was prepared by adding 13.35 ml of 30% (v/v) glycerol solution, supplied in the kit, to the buffer, along with 40 mg/ml of iodoacetamide (alkylating agent). Iodoacetamide was added to the equilibration buffer 2 to prevent sulfhydryl bond formation between free thiol groups of cysteine residues which interfere with the 2D electrophoresis step. Contents were mixed at room temperature using a stir plate until all solids were fully dissolved. 4 ml of equilibration buffer 1 was added to each rehydration/equilibration tray channel containing an IPG strip. The tray was placed on an orbital shaker and gently shaken for 10 min at room temperature. A slow shaker speed was used to prevent the buffer from sloshing out of the tray. At the end of the 10 min incubation, equilibration buffer 1 was discarded by tipping the liquid gently from the tray. Once most of the liquid was decanted the tray was flicked to remove the last drops of equilibration buffer 1. Four milliliters of equilibration buffer 2 were then added to each channel of the rehydration/equilibration tray containing an IPG strip and incubated for 10 min at room temperature with shaking (on an orbital shaker). *Second-dimension (2-D):* the second-dimension step (2D sodium dodecyl sulfate polyacrylamide gel electrophoresis) was performed in 4–15% Criterion™ TGX™ Precast Midi SDS-PAGE gels (11 cm IPG/prep + 1 well) (BioRad, catalogue no. 5671081). Briefly, the IPG strip was rinsed in a graduated cylinder containing 100 ml of 1× Tris/glycine/SDS running buffer. The 4–15% Criterion™ TGX™ Precast Midi SDS-PAGE gel well was rinsed with nanopure water and the excess water blotted using Whatman 3MM paper. The IPG strip was laid gel side onto the back plate of the SDS-PAGE gel. Holding the SDS-PAGE vertically, ReadyPrep Overlay agarose solution (BioRad, catalogue no. 163-2111) was added gently to the well using a Pasteur pipet. Using forceps, the IPG strip was carefully mounted/pushed onto the well, avoiding air bubbles underneath the strip, and the agarose left to solidify for 5 min at room temperature. SDS Proteins were eluted from the strip by adding isoelectric focusing (IEF) gel sample buffer (supplied with the kit) containing 50% (v/v) glycerol (BioRad, catalogue no. 161-0763) and Coomassie blue R-250 stain for sample visualization. Proteins were resolved by electrophoresis in 1× Tris/glycine/SDS running buffer containing (25 mM Tris, 192 mM glycine, 0.1% (w/v) sodium dodecyl sulfate (SDS) at pH 8.3) at 150 V constant for ∼1 h 30 min to 2 h. Proteins were then transferred onto a 0.2 μm nitrocellulose membrane (BioRad, catalogue no. 1620112) for 1 h 30 min at 100 V constant by wet (immersion) transfer in a modified Towbin 1× transfer buffer (25 mM Tris, 192 mM glycine at pH 8.3) (55) containing low (10% (v/v)) methanol and 0.1% (w/v) sodium dodecyl sulfate (SDS) for easier transfer of larger molecular weight proteins. Membrane was blocked in 5% (w/v) skimmed milk in TBS-T for 1 h at room temperature on an orbital shaker, followed by incubation overnight at 4°C on an orbital shaker with primary anti-human LARP1 antibody (Abcam, catalogue no. 86359) at 1:1000 dilution in 5% (w/v) bovine serum albumin (BSA, heat shock fraction) (Millipore Sigma, catalogue no. A7906) in TBS-T. Unbound primary antibody was washed by incubating membranes twice for 5 min each time in TBS-T. The membrane was subsequently incubated with anti-rabbit horseradish peroxidase (HRP)-conjugated IgG (Millipore Sigma, catalogue no. A0545) for 1 h at room temperature on an orbital shaker, at which point the unbound secondary antibody was washed three times in TBS-T (5 min each time). Protein was detected by enhanced chemiluminescence (ECL) using the Western Lightning Plus-ECL reagent (Perkin Elmer Inc., catalogue no. NEL105001EA). ECL signal was detected by autoradiography using HyBlot CL film (Denville Scientific Inc., catalogue no. E3018).

### Orthophosphate labeling

HEK 293T cells were propagated to near-confluency (∼80%) in 10 cm tissue culture-treated polystyrene dishes (Corning, catalogue no. 430167) at 37°C in a humidified incubator at 5% (v/v) CO_2_ in phosphate-containing complete growth media ((DMEM) High Glucose (HyClone GE Healthcare, catalogue no. SH30022.01) supplemented with 10% (v/v) fetal bovine serum (Millipore Sigma, catalogue no. F1051) and 1% (v/v) penicillin/streptomycin (HyClone GE Healthcare, catalogue no. SV30010)). Once cells reached ∼80% confluency, the complete growth media was aspirated and replaced with 5 ml of fresh complete growth media containing phosphorus 32 (^32^P) orthophosphoric acid (Perkin Elmer, NEX053005MC) (∼1 mCi were used per 10 cm dish) in the presence of 0.1% (v/v) DMSO (vehicle), 100 nM rapamycin or 300 nM torin1. Cells were incubated with vehicle/drugs for 3 h at 37°C in a humidified incubator at 5% (v/v) CO_2_. Washed once in ice-cold phosphate buffered saline (PBS) and lysed in 1 ml radio-immunoprecipitation assay (RIPA) extraction buffer containing 50 mM Tris–HCl (pH 8 at room temperature), 150 mM sodium chloride, 1% (v/v) Igepal CA-630 (Nonidep P40, NP40), 0.5% (w/v) sodium deoxycholate and 0.1% (w/v) sodium dodecyl sulfate (SDS), 50 mM sodium fluoride, 1.5 mM sodium orthovanadate, 1 μg/ml RNase A (Millipore Sigma, catalogue no. 10109169001), 1 mM dithiothreitol (DTT) and cOmplete™ Mini EDTA-free protease inhibitor cocktail tablets (Millipore Sigma, catalogue no. 04693159001). Cells were scraped and lysates pre-cleared by centrifugation at 16 000 *g* for 10 min at 4°C. Supernatants were transferred into a fresh microfuge tube and used for radio-immunoprecitation as follows: 900 μl lysate were incubated with 9 μl of anti-human LARP1 rabbit polyclonal antibody (AbCam, catalogue no. ab86359) for 1 h 30 min rotating end-over-end at 4°C. Then added 35 μl of pre-washed protein G-conjugated magnetic Dynabeads (Life Technologies by Thermo Fisher Scientific, catalogue no. 10003D) to the antibody/lysate mixture and incubate for 1 h rotating end-over-end at 4°C. Following the 1 h incubation, the beads were pelleted by centrifugation at 1000 × *g* for 5 min on a tabletop centrifuge at 4°C, the supernatant aspirated and collected for analysis of unbound material. The beads were then washed thrice with 1 ml of RIPA extraction buffer followed by resuspension in 50 μl 4x SDS-PAGE sample buffer and boiling for 5 min at 95°C. Beads were then pelleted and stored at –20°C until further analysis by SDS-PAGE/western blot/^32^P-autoradiography. 10 μl of immunoprecipitate was used to monitor the phosphorylation of endogenous LARP1 by SDS-PAGE/western blot/^32^P-autoradiography. SDS-PAGE/western blot was performed as described above (up until the blocking step). Following blocking the nitrocellulose membrane was enveloped in cling film and exposed for 2–48 h to autoradiography HyBlot CL film (Denville Scientific Inc., catalogue no. E3018) at –80°C and developed using the Konica Minolta Medical and Graphic film processor (Model SRX-101A). The membrane was subsequently rehydrated in TBS-T and used for western blot analysis. Specifically, the membrane was probed with anti-human LARP1 rabbit polyclonal antibody (Abcam, catalogue no. ab86359) in TBS-T for 1 h at room temperature on an orbital shaker, washed twice (5 min each time) with TBS-T and incubated with anti-rabbit horseradish peroxidase (HRP)-conjugated IgG (Millipore Sigma, catalogue no. A0545) for 1 h at room temperature on an orbital shaker, at which point the unbound secondary antibody was washed three times in TBS-T (5 min each time). Protein was detected by enhanced chemiluminescence (ECL) using the western lightning plus-ECL reagent (Perkin Elmer Inc., catalogue no. NEL105001EA). ECL signal was detected by autoradiography using HyBlot CL film (Denville Scientific Inc., catalogue no. E3018). Samples of lysates (5 μl) were also analyzed by SDS-PAGE/western blot for mTORC1 activation using phospho-specific antibodies against T389 on S6K1 and T37/T46 on 4E-BP1 as described in the SDS-PAGE/western blot materials and methods section.

### mTORC1 *In Vitro* kinase assays

HEK 293T CRISPR/Cas9 LARP1^KO^ cells were transfected with 10 μg pRK5 empty vector or 5 μg pRK5 Myc-mTOR and 5 μg pRK5 HA-RAPTOR as described above. Cells were stimulated with complete growth media for 3 h before cell lysates were prepared. 800 μl lysate was added to each protein-IP using 5.5 μl anti-HA antibodies to pull down the mTOR-RAPTOR complex as described above for protein-IPs. After washing, the immunoprecipitated bead-complexes remained in a volume of 10 μl. Bead-complexes were pretreated for 35 min at room-temperature with DMSO, rapamycin/GST-FKBP12 or torin1 in a total volume of 55 μl of reaction buffer 31.8 mM HEPES pH 7.5, 63.6 mM KCl, 12.7 mM MgCl_2_, 2.5 mM MnCl_2_, 0.64 mM DTT) supplemented with either 0.64% (v/v) DMSO, 545 nM rapamycin (+500 ng GST-FKBP12) or torin1 (164 nM). Bead-complexes were then added 15 μl LARP1-mix to a reaction volume of 70 μl containing 5.6 μg Myc/FLAG/6xHis-LARP1 protein, 25 mM HEPES pH 7.5, 50 mM KCl, 10 mM MgCl_2_, 2 mM MnCl_2_, 0.5 mM DTT, 0.14 mM ATP, 14 μCi γ-^32^P–ATP and drugs at a final concentration of 0.5% (v/v) DMSO, 429 nM rapamycin (+500 ng GST-FKBP12) or 129 nM torin1. The *in vitro* kinase reaction was run for 30 min at 30°C and 1400 rpm after which the reaction was stopped by adding 25 μl 5× SDS sample buffer and boiling 7 min at 95°C. Proteins were separated by SDS-PAGE and transferred to a nitrocellulose membrane. Phosphorylation was monitored by autoradiography and total protein levels determined by western blotting.

### Mass spectrometry data handling

The raw data for the rapamycin screen were extracted from the previous study (52). The raw MS/MS spectra were searched against a composite database of the mouse IPI protein database and its reversed complement using the Sequest algorithm. Search parameters include a static modification of 57.02146 Da for Cys, and a dynamic modification of phosphorylation (79.96633 Da) on Ser, Thr and Tyr. Furthermore, a dynamic modification was also considered for oxidation (15.99491 Da) on Met, and stable isotope (10.00827 Da) and (8.01420 Da) on Arg and Lys, respectively. Search results were filtered to a 1% false-discovery rate (FDR) using the linear discriminator function (57). Phosphorylation site localization was assessed by the ModScore algorithm and peptide quantification was performed by using the CoreQuant algorithm (57).

### RNA-electrophoretic mobility shift assay (RNA-EMSA)

RNA-EMSAs were performed and imaged as reported previously using the same amount of RNA (≤200 pM) regardless of labeling efficiency (28). All RNAs were snap-cooled by heating at 95°C in 1× binding buffer for 1 minute and immediately transferred to ice for 20 min. 5× protein stocks were prepared in protein dilution buffer (50 mM Tris–HCl, pH 7.5, 250 mM NaCl, 25% glycerol, 2 mM DTT). 10 μl reactions contained 2 μl 5× protein stock, 2 μl 5× RNA stock at 20 nM, 2 μl 5× binding buffer, resulting in final concentrations of 20 mM Tris–HCl, pH 8, 150 mM NaCl, 10% glycerol, 1 mM DTT, 0.5 μg tRNA and 1 μg BSA. Reactions were incubated on ice for 30 min and 8 μl were loaded on 7–8% polyacrylamide (29:1) native 0.5× TBE gels at 4°C. Gels were run at 120 V for 40 min, dried, and exposed overnight. Phosphor screens (GE Lifesciences) were imaged on a Typhoon FLA plate reader (GE Lifesciences) and quantitated using Imagequant TL (GE Lifesciences).

### Protein expression and purification

Plasmids expressing mutants of DM15 were generated using site-directed mutagenesis and confirmed by Sanger sequencing. The LARP1 coding sequence (amino acids 796–946 from LARP1) was cloned by PCR from the full-length LARP1 coding sequence into a modified pET28a vector. The resulting construct expressed DM15 with an N-terminal His_6_-MBP tag followed by a tobacco etch protease cleavage site and glycine_6_ linker. This expression plasmid was transformed into BL21(DE3) *E. coli* cells and grown overnight on LB agar plates supplemented with 30 μg/ml kanamycin. The His_6_-MBP-DM15 fusion protein was expressed by autoinduction for 3 h at 37°C and at 18°C for 18 h. Cells were collected by centrifugation, flash frozen in liquid nitrogen, and stored at –80°C until used. 2 g of cells were resuspended by gentle stirring in NiNTA lysis buffer (50 mM Tris–HCl, pH 7.5, 400 mM NaCl, 10 mM imidazole, 10% glycerol) for 1 h with protease inhibitor cocktail (PMSF, Leupeptin, Bestatin and Aprotinin). Cells were lysed by homogenization and clarified by centrifugation at 12 000 RPM at 4°C for 30 min. The soluble fraction was nutated with 4 ml HisPur Ni-NTA Resin (ThermoFisher product 88221) for 2 h at 4°C. The beads were washed 2 times in 50 ml lysis buffer and 3 times with wash buffer (50 mM Tris–HCl, pH 7.5, 400 mM NaCl, 35 mM imidazole, 10% glycerol). His_6_-MBP-(665–947) fusion protein was eluted from beads in 15 ml elution buffer (50 mM Tris, pH 7.5, 400 mM NaCl, 250 mM imidazole, 10% glycerol). The N-terminal His_6_-MBP tag was removed by the addition of 2 mg tobacco etch protease for cleavage overnight. Cleaved DM15 protein was further purified of RNA and protein contaminants by tandem HiTrap Q and HiTrap SP columns (GE Lifesciences). DM15 protein free of nucleic acid contaminants was eluted from the HiTrap SP column with gradient from 150 mM NaCl to 1 M NaCl over 50 ml. MBP flowed through both columns while untagged DM15 eluted at 35% elution buffer. Remaining uncleaved fusion protein eluted at 20% elution buffer, allowing for efficient separation of cleaved DM15. Fractions containing DM15 were pooled and brought to 1 M ammonium sulfate by the dropwise addition of 3M ammonium sulfate with gentle swirling. The protein was diluted to 40 ml in 50 mM Tris–HCl, pH 7, 1 M ammonium sulfate and loaded onto a 5 ml Butyl HP column (GE Lifesciences) at 0.5 ml/min. The Butyl HP column was eluted over 5 CV in 50 mM Tris–HCl, pH 7, 2 mM DTT. The fractions containing DM15 were collected, concentrated, and buffer exchanged with a 10K MWCO Amicon Ultra spin concentrator (Millipore) into 25 mM Tris–HCl, pH 7.5, 2 mM DTT, 250 mM NaCl and loaded on an equilibrated GE HiLoad 16/600 Superdex 75 gel filtration column (GE Lifesciences) run at 1 ml/min. DM15-containing fractions were pooled and glycerol increased to 20% before being flash frozen in 10 μl aliquots and stored at –80°C for further use.

### Statistical analysis

**Figure 1.**
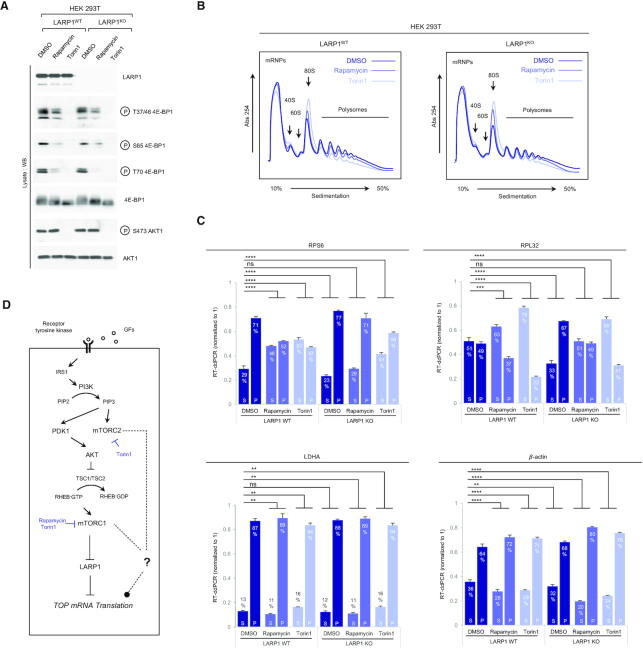
Rapamycin and Torin1 repress translation of TOP mRNAs *via* LARP1. (**A**) HEK293T CRISPR/Cas9 LARP1 wildtype (WT) or knockout (KO) cells (clone 5, *cf*. Supplementary Figure S1) were cultured to ∼70–80% confluency at which point they were replenished with complete growth media (containing 10% (v/v) fetal bovine serum) containing either 0.1% (v/v) DMSO (vehicle), 100 nM Rapamycin or 300 nM Torin1, for 3 h. Cells were lysed in hypotonic buffer in the presence of RNase inhibitors and samples of lysates were analyzed by SDS-PAGE/Western blot for activation of mTORC1 and mTORC2 using the indicated antibodies. (**B**) Samples of lysates were subjected to sucrose gradient ultracentrifugation/polysome profile analysis as follows: lysates were quantified for total RNA (predominantly rRNA) concentration by monitoring absorbance at 260 nm. Equal amounts of total RNA were loaded onto each sucrose gradient (total volume adjusted in hypotonic buffer) and subjected to ultracentrifugation as detailed in the *Experimental Procedures* section. Polysome profile traces (absorbance 254 nm) are shown. Total RNA was extracted from each polysomal fraction and pooled into subpolysomal (S) and polysomal (P). cDNA was synthesized by reverse-transcription (RT) and abundance of specific transcripts analyzed by digital droplet PCR (ddPCR) as described in further detail in the *Experimental Procedures* section. (**C**) *Bar graph* data displaying percentage abundance of each TOP (RPS6, RPL32) and non-TOP (LDHA, *β*-actin) transcript in S and P fractions. *Error bars* denote standard deviation (St. Dev.) for three technical replicates. Refer to *Statistical workbook* for raw data and statistical analysis. (**D**) Diagram depicting the PI3K–AKT–mTORC1–LARP1 signalling pathway and target sites of pharmacological inhibitors.

**Figure 2.**
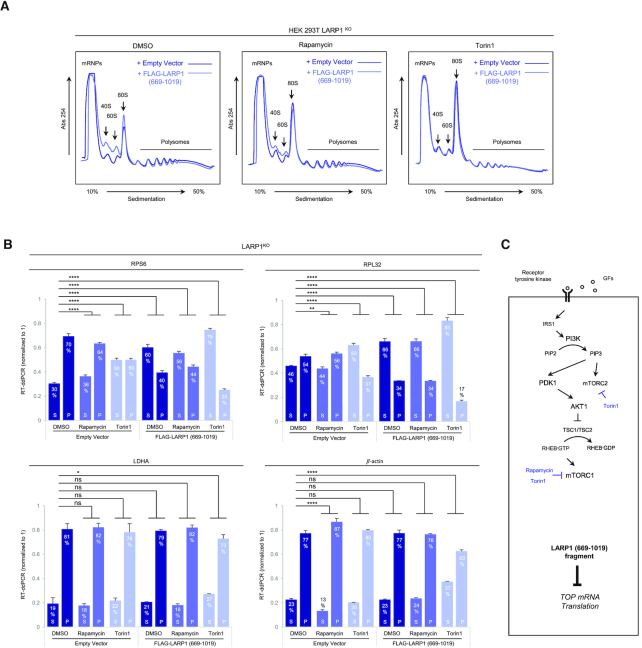
Overexpression of a C-terminal fragment of LARP1 (spanning residues 669–1019) constitutively represses TOP mRNA translation in an mTORC1-independent manner. (**A**) HEK293T cells were cultured to near-confluency (∼70–80%) at which point cells were replenished with fresh complete media (containing 10% (v/v) fetal bovine serum) containing either 0.1% (v/v) DMSO (vehicle), 100 nM rapamycin or 300 nM Torin1 for 3 h. Cells were lysed in hypotonic buffer and samples of lysates were subjected to sucrose gradient ultracentrifugation/polysome profile analysis. Absorbance (254 nm) profiles are shown. (**B**) Total RNA was extracted from each fraction and pooled into subpolysomal (S) and polysomal (P) fractions. cDNA was synthesized from S and P fractions by reverse-transcription (RT) and specific transcripts quantified by droplet digital PCR (ddPCR). Data shown as percentage abundance on S and P fractions. *Error bars* denote standard deviation (St. Dev.) for three technical replicates. Translation efficiency was determined as the ratio of P/S. (**C**) Diagrammatic representation of PI3K/AKT/mTORC1 pathway and ability of the cap-binding fragment of LARP1 to constitutively repress TOP mRNA translation.

Data shown within this manuscript were derived from representative experiments. Two or more experimental replicates were performed from representative experiments. Error bars in *bar graphs* in Figures 1C, 2B, 10C, D, 11C denote standard deviations (SD) for three technical replicates within each experiment. Statistical analyses were performed using Prism 5 (GraphPad Software, California). One-way and two-way analysis of variance (ANOVA) with Dunnett's post-hoc tests were performed in Figures 10, 11 and Figures 1, 2, respectively. Legend: *P < 0.05, **P < 0.01, ***P < 0.001, ****P < 0.0001, ‘ns’ denotes non-significant.

## RESULTS

### mTORC1 controls TOP mRNA translation *via* LARP1

Recently, we (21) and others (26) used RNA interference (RNAi) technology to demonstrate that mTORC1 controls TOP mRNA translation through LARP1. RNAi leads to incomplete depletion of LARP1 and as a technology is limited in its scope. To fully grasp the contribution of LARP1 to the control of TOP mRNA translation, we generated HEK 293T LARP1 knockout (LARP1^KO^) clonal cell lines *via* CRISPR/Cas9 genomic editing (Supplementary Figure S1A–D) and monitored polysome association of representative TOP (ribosomal protein S6 [RPS6] and ribosomal protein L32 [RPL32]) and non-TOP (lactate dehydrogenase A [LDHA] and β-actin [beta-actin]) transcripts (Supplementary Figure S2A, B). Specifically, we monitored polysome distribution for TOP and non-TOP transcripts (in LARP1^WT^ and LARP1^KO^ cell lines) in conditions of acute (i) mTORC1 activation, (ii) mTORC1 inactivation or (iii) dual mTORC1/mTORC2 inactivation. This was accomplished by culturing each cell line in complete growth media for 3 h in the presence of either (i) DMSO (mTORC1 activation), (ii) rapamycin (mTORC1 inactivation) or (iii) torin 1 (dual mTORC1/mTORC2 inactivation).

Acute stimulation of LARP1^WT^ and LARP1^KO^ cells with complete growth media leads to activation of both mTORC1 and mTORC2 in both cell types to identical extents (Figure 1A; DMSO), indicating that, contrary to an earlier report (53), the absence of LARP1 does not alter the activation status of the mTOR complexes in our hands. Absence of LARP1 also does not alter the sensitivity of other mTOR targets to rapamycin or torin1 (Figure 1A). Cellular extracts from LARP1^WT^ and LARP1^KO^ cells in Figure 1A were then subjected to polysome profile analysis (Figure 1B). Treatment with rapamycin leads to a subtle (but reproducible) decrease in polysome abundance and a concomitant increase in monosome (80S) abundance in both LARP1^WT^ and LARP1^KO^ cells to similar extents. These data suggest that rapamycin weakly reduces global protein synthesis to similar extents in both cell lines. Torin1 also inhibits global protein synthesis albeit more strongly than rapamycin does (Figure 1B), likely reflecting the more potent inhibitory effect of torin1 on the mTORC1 complex coupled to its ability to block the activity of both mTORC1 and mTORC2 (58). Treatment with torin1 leads to a marked reduction in polysome abundance, an increase in 80S absorbance, and a slight (but reproducible) decrease in the formation of mRNA ribonuclear particles (mRNPs) (Figure 1B). Importantly, torin1 appears to block global protein synthesis to similar extents in LARP1^WT^ and LARP1^KO^ cells (Figure 1B).

We then compared polysome association of TOP and non-TOP mRNAs in LARP1^WT^ and LARP1^KO^ cells in conditions of mTORC1/mTORC2 activation/inactivation (Figure 1C). As expected, we observed that in conditions of mTORC1 activation (DMSO), genetic deletion of *LARP1* leads to an accumulation of TOP transcripts in polysomal fractions: 71% of RPS6 transcript is typically found polysomal fractions (P) in HEK 293T LARP1^WT^ cells; 77% of this same transcript is found in the P fraction in LARP1^KO^ cells (*P* < 0.0001). Genetic deletion of *LARP1* has an even more pronounced effect on the polysomal association of RPL32 mRNA (the percentage of this transcript in the P fraction is 49% in LARP1^WT^ cells and 67% in LARP1^KO^ cells, *P* < 0.0001) (Figure 1C). By contrast, genetic deletion of *LARP1* does not enhance the polysomal association of LDHA mRNA (*P* = 0.6037, and has only a minor effect on the polysomal distribution of β-actin mRNA (barely significant, *P* = 0.0078) (Figure 1C). These data suggest that LARP1 preferentially represses the translation of TOP mRNAs.

The *LARP1* status of the cell also influences the sensitivity of the translation of TOP transcripts to mTOR inhibitors: we observed that treatment of LARP1^WT^ cells with mTOR inhibitors causes an accumulation of TOP transcripts in poorly translated subpolysomal (S) fractions (e.g. 71% of RPS6 transcript is found in the P fraction in DMSO-treated LARP1^WT^ cells compared to 52% in rapamycin-treated and 47% in torin1 treated LARP1^WT^ cells [*P* < 0.0001 in both cases]) (Figure 1C). Expectedly, torin1 leads to a stronger inhibitory effect on TOP mRNA translation than rapamycin does (*cf*. for example, 37% of RPL32 transcript in the P fraction in rapamycin-treated LARP1^WT^ cells compared to 22% in torin1-treated LARP1^WT^ cells). By contrast, the translation of non-TOP transcripts in LARP1^WT^ cells is either largely unaffected by acute treatment (3 h) with mTOR inhibitors (e.g. LDHA) or, as is the case of β-actin mRNA, enhanced by these drugs (8% increase in β-actin transcript in the P fraction upon rapamycin treatment) (Figure 1C).

Importantly, genetic deletion of *LARP1* markedly alters the sensitivity of TOP mRNA translation to mTOR inhibitors but not that of non-TOP mRNAs (Figure 1C). For example, rapamycin causes a statistically significant shift of the RPS6 transcript from polysomal (P) to subpolysomal (S) fractions in LARP1^WT^ cells (from 71% to 52%, *P* < 0.0001) while, the effect of rapamycin on RPS6 translation in LARP1^KO^ cells is not statistically significant (from 77% to 71%, *P* = 0.9972) (Figure 1C, see also the Statistical Workbook). Similarly, torin1 potently represses TOP mRNA translation in LARP1^WT^ cells and less efficiently so in LARP1^KO^ cells. Similar results were observed for the RPL32 transcript: while rapamycin markedly reduces the association of RPL32 mRNA with polysomes in LARP1^WT^ cells (as noted by the decrease in transcript levels in the P fraction [49% to 37%, *P* = 0.0003]), it fails do so in LARP1^KO^ cells (transcript levels in P fraction remain the same [49%, *p* = non-significant]) (Figure 1C). Torin1 also reduces the amount of RPL32 transcript in the P fraction in LARP1^KO^ cells, but it does so less effectively than in LARP1^WT^ cells (Figure 1C). Collectively, these data corroborate our original model that LARP1 functions as a repressor of TOP mRNA translation downstream of mTORC1 (21). It is important to note that deletion of *LARP1* fails to completely de-repress the inhibitory effects of both rapamycin and torin1 on TOP mRNA translation (Figure 1C), indicating that additional, presently unknown, mTORC1/mTORC2-dependent (but LARP1-independent) mechanisms of TOP mRNA translation repression likely exist (Figure 1D).

### The DM15 region constitutively represses the translation of TOP mRNAs, even in conditions of full mTORC1 activation

We determined that the DM15 region of LARP1 binds the m^7^Gppp and adjacent 5′TOP motif of TOP mRNAs (23); in doing so, LARP1 efficiently displaces eIF4E and eIF4G1 from TOP mRNAs (21,23). An extended version of the LARP1 C-terminus containing the DM15 domain has also been shown to block cap-dependent translation of TOP transcripts in a reporter assay (24). These observations raised the intriguing possibility that the DM15 domain mediates the translation repression effect of LARP1 on TOP mRNA translation. To test this, we transiently re-expressed a C-terminal fragment of LARP1 (residues 669–1019) encompassing the entire C-terminal half of LARP1 that includes the DM15 region in LARP1^KO^ cells, followed by acute (3 h) exposure to complete growth media and either DMSO (vehicle) or mTOR inhibitors. Lysates were then subjected to polysome profiling (Figure 2A). Overexpression of the C-terminal fragment caused a small (but reproducible, see also Figure 11B) increase in the 40S, 60S and 80S peaks in polysome profiling traces of DMSO-treated cells, suggesting that overexpression of the DM15 region causes a reduction in global protein synthesis in growth conditions. Rapamycin causes similar increases in 40S, 60S and 80S peaks in a similar manner to overexpression of the LARP1 (669–1019) fragment (Figure 2A). Torin1 treatment recapitulates the effects of LARP1 (669–1019) fragment overexpression, such that the two polysome profile traces become superimposable (Figure 2A).

Having established that re-expression of the LARP1 (669–1019) fragment likely impacts global protein synthesis, we then assessed its effect on the translation of TOP transcripts. We performed RT-ddPCR analysis to assess the distribution of TOP and non-TOP transcripts across the gradient: like in Figure 1C, translation of TOP mRNAs is elevated in LARP1^KO^ cells (Figure 2B), and rapamycin and torin1 fail to strongly supress TOP mRNA translation in the absence of LARP1 (Figure 2B); notably, re-introduction of the C-terminal fragment of LARP1 restores TOP mRNA translation inhibition, even in the conditions of growth stimulation (DMSO, vehicle) (Figure 2B). Re-expression of the C-terminal fragment of LARP1 leads to a 30% decrease of RPS6 mRNA in the P fraction of DMSO treated cells (from 70% to 40%, *P* < 0.0001) (Figure 2B). A similar inhibitory effect was observed for RPL32 mRNA polysomal distribution: re-expression of the C-terminal fragment of LARP1 resulted in a 17% decrease of RPL32 mRNA in the P fraction of DMSO-treated cells (from 54% to 37%, *P* < 0.0001) (Figure 2B). Importantly, the C-terminal fragment of LARP1 did not repress the translation of non-TOP transcripts (LDHA and β-actin) (Figure 2B). Our observations that the C-terminal LARP1 fragment represses translation of TOP mRNAs (but not that of non-TOP transcripts) in conditions of mTORC1 activation and inactivation demonstrate that: (i) the C-terminal LARP1 fragment selectively inhibits the translation of TOP transcripts and (ii) that it acts as a constitutive TOP mRNA translation repressor effectively decoupling mTORC1 from TOP mRNA translation (Figure 2B,C).

### mTORC1 interacts primarily with LARP1 (among LARP superfamily members)

**Figure 3.**
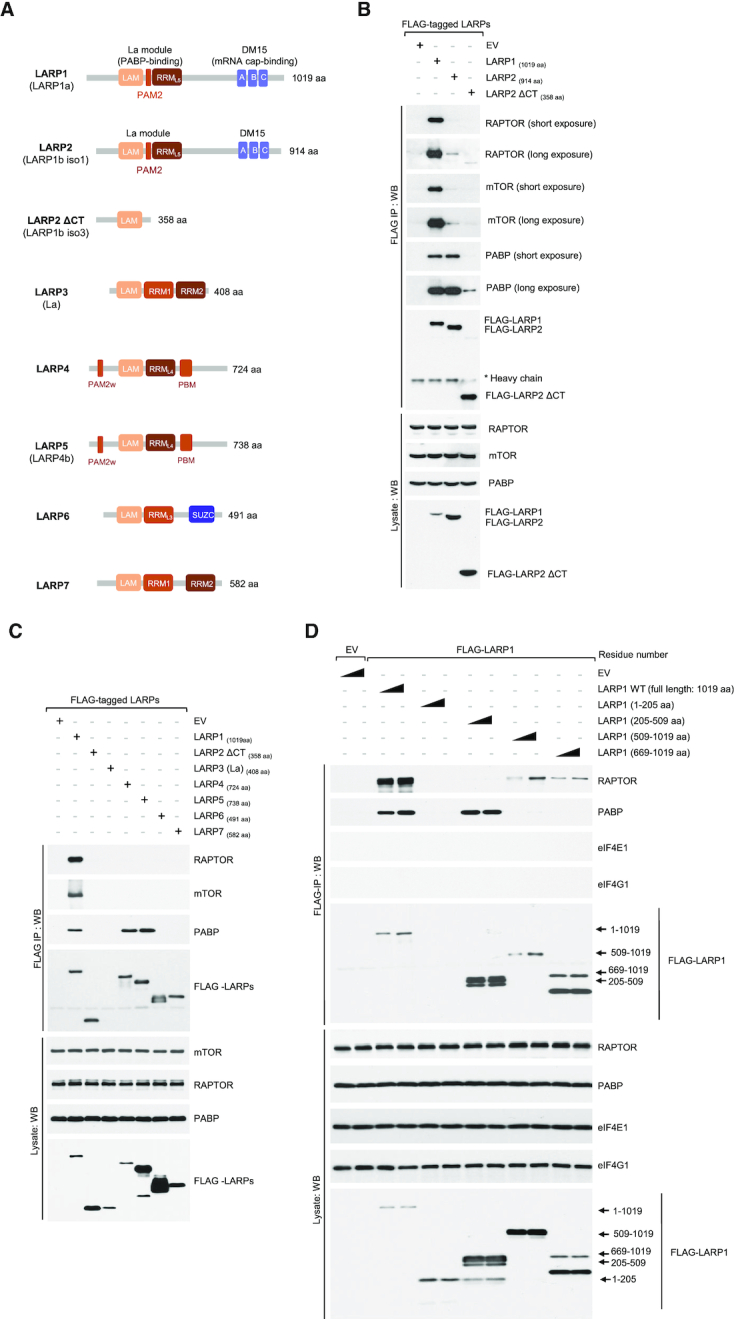
LARP1 and LARP2 bind PABP *via* the La module, while only LARP1 binds mTORC1 *via* the DM15 region. (**A**) Diagrammatic representation of the human LARP superfamily. (**B**) LARP1 and LARP2 interact with PABP, while only LARP1 binds mTORC1. HEK 293T cells were transiently transfected with empty vector (EV), FLAG-tagged LARP1, –LARP2 or a variant of LARP2 that lacks the C-terminal region (–LARP2 ΔCT). Cells were stimulated with full growth media for 3 h and lysed in CHAPS-based extraction buffer (see *Experimental Procedures* section). Lysates were subjected to immunoprecipitation (IP) and analyzed by SDS-PAGE/western blot with the indicated antibodies. (**C**) LARP1, LARP2, LARP4 and LARP5 interact with PABP. HEK 293T cells were transfected and treated as described in (B) and lysates subjected to IP with anti-FLAG antibody. (**D**) RAPTOR binds to the C-terminal region of LARP1 that comprises the DM15 domain, while PABP binds to the La module that comprises the previously characterized PAM2 motif. LARP1 does not interact with eIF4G1 or eIF4E1. HEK 293T cells were transfected with empty vector (EV), full length FLAG-tagged wildtype LARP1 (WT) or FLAG-tagged fragments of LARP1. Amino acid numbering indicated. Transfected cells were treated and lysed as described in (A). Lysates were subjected to IP with anti-FLAG antibody and analyzed by SDS-PAGE/western blot with the indicated antibodies. Triangle denotes plasmid DNA concentration

We have previously shown that LARP1 interacts with mTORC1 *via* the scaffolding protein RAPTOR (short for regulatory associated protein of mTOR), specifically through contact points with the RNC (RAPTOR N-terminal conserved) region and the WD40 (tryptophan/aspartate repeats 40 amino acids-long) domain (21). Given the high level of sequence conservation between the human LARP1 and LARP2 (also known as LARP1b) proteins (60% identity and 73% similarity at amino acid level) (Figure 3A and Supplementary Figure S3), we asked whether LARP2 also interacts with mTORC1. HEK 293T cells were transiently transfected with FLAG-tagged human LARP1, human LARP2 or a variant of human LARP2 that lacks the C-terminal region (human LARP2 ΔCT) (Supplementary Figure S4), cells were stimulated with complete growth media and lysed in CHAPS-based buffer that preserves protein-protein interaction; lysates were subjected to immunoprecipitation with anti-FLAG tag antibody and probed for endogenous RAPTOR and endogenous mTOR proteins by western blot. The resulting data revealed that LARP1 (in this particular case exogenous FLAG-LARP1) interacts strongly with both RAPTOR and mTOR (Figure 3B). By contrast, LARP2 interacts weakly with RAPTOR and mTOR (Figure 3B). Both FLAG-LARP1 and FLAG-LARP2 interact with PABP to similar extents (Figure 3B), indicating that the weak association of LARP2 with mTORC1 is not the result of protein misfolding, as it maintains the capacity to interact with other proteins. The FLAG-LARP2 ΔCT variant which lacks the PAM2 motif (as well as the RRM_L5_ and the DM15 region) exhibits reduced PABP-binding (Figure 3B), consistent with our earlier observation that the PAM2 motif in LARP1 mediates binding to PABP (21). We extended this analysis to other LARP superfamily members (Figure 3C). As observed for LARP2: La, LARP4, LARP5, LARP6 and LARP7 failed to associate with RAPTOR (Figure 3C). Collectively, these data show that LARP1 is the sole member of this superfamily of proteins that is capable of interacting strongly with mTORC1.

### LARP1 interacts with mTORC1 *via* the carboxy-terminal region that comprises the DM15 motif

Next, we assessed which regions of LARP1 mediate interactions with mTORC1. We overexpressed FLAG-tagged full-length human LARP1 or fragments of human LARP1 covering various regions of the protein. Full-length LARP1 efficiently co-precipitates endogenous RAPTOR (and PABP but not eIF4E or eIF4G1) (Figure 3D). Fragments 1–205 (comprising the N-terminal region of LARP1) and 205–509 (comprising the mid-region of LARP1 that mediates PABP binding) do not co-precipitate RAPTOR. Only the C-terminal fragments co-precipitate RAPTOR (and rather weakly) (Figure 3D), indicating that RAPTOR interacts with multiple regions of LARP1, but primarily through the C-terminal region that includes the DM15 motif. The poor binding of this C-terminal fragment of LARP1 to RAPTOR likely explains why this same fragment retains its translation inhibitory ability in conditions of mTORC1 activation, i.e. this fragment interacts rather weakly with mTORC1 and as such is most likely poorly phosphorylated by mTORC1. In this case, it would remain bound to the 5′TOP sequence and constitutively repress TOP mRNA translation even in conditions of mTORC1 activation (Figure 2B).

**Figure 4.**
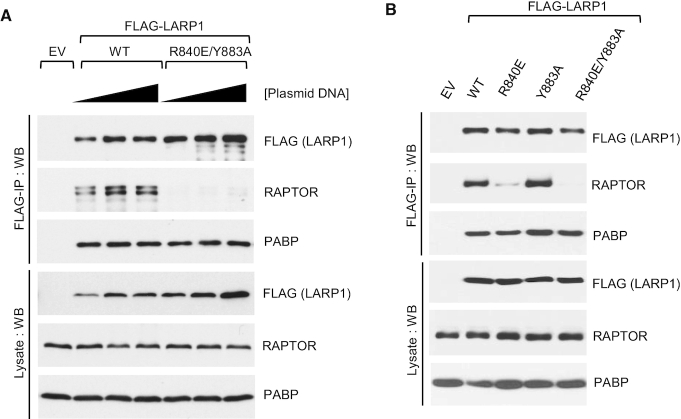
Residue R840 in LARP1 is required for RAPTOR binding. (**A**, **B**) HEK293T cells were transiently transfected with empty vector (EV), human FLAG-tagged wildtype LARP1 (1019aa isoform) or the R840E/Y883A double-mutant. Cells were stimulated with complete growth media for 3 h and then lysed in CHAPS-based extraction buffer in the presence of RNase A. Lysates were subjected to immunoprecipitation with anti-FLAG antibody and analysed by SDS-PAGE/Western blot using the indicated antibodies.

In our quest to identify the contact points between LARP1 and RAPTOR, we looked further into specific residues within the DM15 motif. We have previously shown that the R840 and Y883 residues plays critical roles in guiding the 5′TOP sequence through a positively charged channel within the DM15 domain and binding the m7Gppp cap structure, respectively (23). Fortuitously, we observed that the LARP1 R840E/Y883A double mutant exhibits markedly reduced binding to RAPTOR compared to wildtype LARP1 (Figure 4A). We sought to investigate whether R840, Y883 or both residues are involved in mediating RAPTOR binding. To this end, we generated single amino acid substitutions for each of these residues. Single amino acid analysis revealed that R840 (and not Y883) is the key residue mediating the association of LARP1 with RAPTOR, in that a charge reverse of R840 to a glutamate suffices in reducing RAPTOR binding (Figure 4B). By contrast, substitution of Y883 for alanine did not affect RAPTOR binding. Amino acid R840 is, however, likely insufficient for the binding of LARP1 to mTORC1, since R840 is conserved in LARP2 (Supplementary Figure S3) yet this protein does not interact with mTORC1 (Figure 3B).

**Figure 5.**
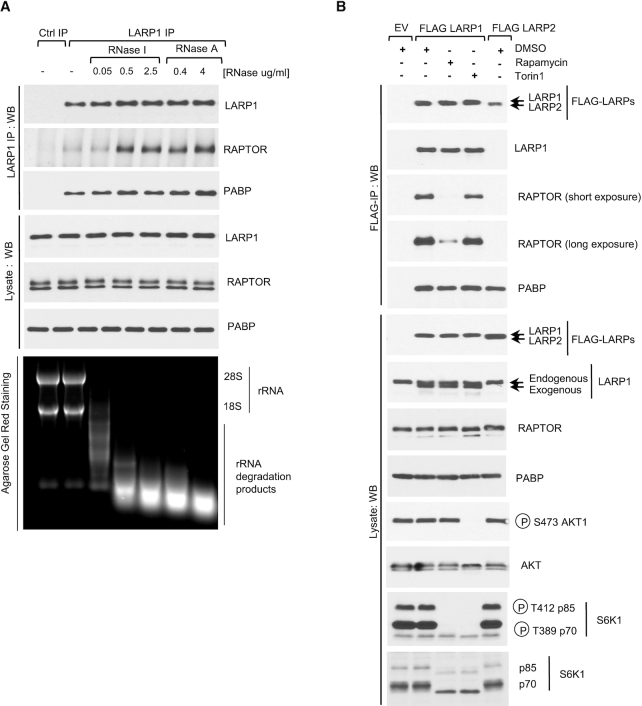
Ribonuclease treatment enhances the binding of LARP1 to RAPTOR. Rapamycin but not Torin1 dissociates LARP1 from RAPTOR. (**A**) RNase A and RNase I enhance the interaction between endogenous LARP1 and mTORC1. HEK293T cells were stimulated with complete media (experimental procedures) for 3 h and then lysed in CHAPS-based extraction buffer in the presence or absence of RNase A or RNase I in various concentrations. Lysates were subjected to immunoprecipitation with anti-LARP1 antibody and were analyzed by SDS-PAGE/western blot using the indicated antibodies. Agarose Gel Red Staining was used to monitor rRNA integrity. (**B**) HEK293T cells were transfected with FLAG-tagged LARP1 or LARP2. Where indicated cells were stimulated with full-growth media in the presence of 0.1% (v/v) DMSO (vehicle), 100 nM rapamycin or 300 nM torin1 for 3 h. Cells were lysed as described in (A) and lysates subjected to immunoprecipitation with anti-FLAG antibody.

Previously, we showed that LARP1 interacts with RAPTOR in an RNA-sensitive manner: digestion of RNA with ribonuclease A (RNase A) enhances the binding of LARP1 to RAPTOR (21). Although the mechanism underlying this effect is presently not known, the observation that RNase A enhances LARP1-RAPTOR interaction is reproduced systematically (Figure 5A) and has been corroborated by others research groups (53). Previously, we also showed that, by contrast, the association of LARP1 with PABP is not influenced by RNase A (21); see also Figure 5A. These data suggest that LARP1 interacts directly with PABP, without the requirement for an intact poly(A) tail (21). Hong and colleagues (53) have challenged the concept that LARP1 interacts directly with PABP, claiming that RNase A forms spurious aggregates which lead to non-specific co-precipitation of LARP1 and PABP. We considered it to be important to verify this claim; to this end, we performed LARP1 immunoprecipitation experiments in the presence of an alternative ribonuclease, RNase I. As observed for RNase A, digestion of RNA with RNase I enhances the binding of endogenous LARP1 to endogenous RAPTOR (Figure 5A). Importantly, as previously observed for RNase A (21), RNase I does not affect PABP binding, indicating that the LARP1-PABP interaction does not require an intact poly(A) tail (Figure 5A). The latter result is particularly important because ribonuclease (RNase A) preferentially cleaves pyrimidines-rich sequences and does not digest adenylate polymers efficiently (59). Collectively, these data confirm earlier reports and show that LARP1 interacts directly with PABP (60).

### Rapamycin, but not torin1, disrupts the binding of LARP1 to mTORC1

Next, we tested whether pharmacological inhibition of mTORC1 plays a role in regulating the interaction between LARP1 and mTORC1. HEK 293T cells were transiently transfected with FLAG-LARP1 or FLAG-LARP2 and then stimulated with growth media in the presence of DMSO (vehicle), rapamycin and its binding partner FKBP12 (FK506-binding protein 12), or torin1; lysates were subjected to immunoprecipitation with anti-FLAG antibody and immunoprecipitates analyzed by western blot. As shown in Figure 5B, rapamycin efficiently disrupts the interaction between LARP1 and RAPTOR (but not that of LARP1 with PABP). In parallel, we also tested the effect of torin1 on the interaction between mTORC1 and LARP1. Surprisingly, torin1 does not affect LARP1 association with mTORC1 (Figure 5B), suggesting that the rapamycin-mediated disruption of the LARP1-RAPTOR interaction likely arises from an allosteric effect of rapamycin/FKBP12 on the stability of the mTORC1 complex (61), rather than from a shut-off of mTOR catalytic activity.

### LARP1 is phosphorylated directly by mTORC1 in a rapamycin- and Torin1-Dependent manner

**Figure 6.**
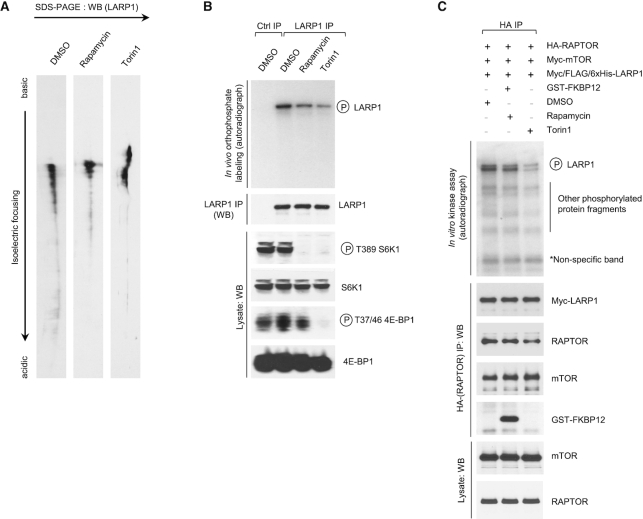
mTORC1 phosphorylates LARP1 *in vivo* and *in vitro* in a rapamycin- and Torin1-dependent manner. (**A**) Isoelectric focusing of endogenous human LARP1 from HEK293T cells stimulated with complete media for 3 h in the presence of 0.1% (v/v) DMSO (vehicle), 100 nM rapamycin or 300 nM Torin1. (**B**) Orthophosphate labeling of HEK293T cells stimulated with complete media for 3 h in the presence of 0.1% (v/v) DMSO (vehicle), 100 nM rapamycin or 300 nM Torin1. Cells were lysed and endogenous LARP1 immunoprecipitated with anti-LARP1-specific antibody as described in the *Experimental Procedures* section. Immunoprecipitates and lysates were analysed by SDS-PAGE/western blot and autoradiography. (**C**) mTORC1 *in vitro* kinase assay. Myc/FLAG/6xHis-tagged human LARP1 (isoform 1096aa) was expressed in insect cells and purified as detailed in the *Materials and Methods* section and was then incubated with HA-RAPTOR/Myc-mTOR immunoprecipitates in the presence of gamma-32P[ATP] in the presence of DMSO, rapamycin and GST-FKBP12 or Torin1. ^32^P-incorporation was monitored by SDS-PAGE/western blot autoradiography. Immunoprecipitates and lysates were analyzed by SDS-PAGE/western blot with the indicated antibodies.

Using a number of complementary biochemical assays, next we investigated whether mTORC1 phosphorylates LARP1. We began by investigating the effects of rapamycin and torin1 on the electric charge of endogenous LARP1 by isoelectric focusing. mTOR inhibitors raise the isoelectric point (p*I*) of LARP1, denoting a reduction in negative charge of LARP1 and possibly reflecting a decrease in phosphorylation (Figure 6A). To confirm that the changes in electric charge reflect changes in phosphorylation (and not other post-translation modifications), we monitored the effect of rapamycin and torin1 on the phosphorylation of LARP1 by *in vivo* orthophosphate metabolic labelling (Figure 6B). Treatment with rapamycin and torin1 decreases the phosphorylation of endogenous LARP1 (Figure 6B) confirming that, indeed, mTORC1 activity influences LARP1 phosphorylation status.

It remained possible that mTORC1 influenced the phosphorylation status of LARP1 indirectly (i.e. *via* a downstream kinase). To confirm that LARP1 is a *bona fide* mTORC1 substrate, we performed *in vitro* mTORC1 kinase assays with [γ-^32^P]-ATP label and recombinant purified myc-6xHis-LARP1 (full-length isoform 1096aa) in the presence of DMSO (vehicle), rapamycin/GST-FKBP12, or torin1. As shown in Figure 6C, mTORC1 directly phosphorylates LARP1 *in vitro*. LARP1 phosphorylation is diminished by rapamycin, and to a larger extent, by torin1. These results confirm that LARP1 is, indeed, a direct mTORC1 target.

### Rapamycin modulates the phosphorylation of 26 serine and threonine and 3 tyrosine residues broadly distributed into 7 clusters

**Figure 7.**
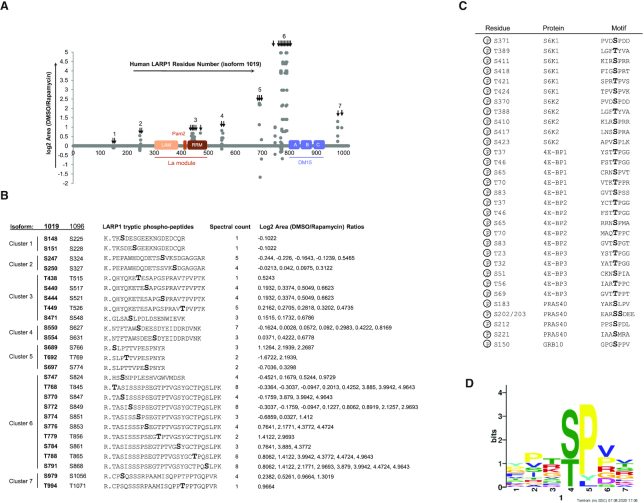
Phosphoproteomic analysis of LARP1 in MEFs display that LARP1 is phosphorylated at upwards of 26 serine and threonine residues, which are broadly grouped into 7 cluster regions in a rapamycin-sensitive manner. (**A**) Graphical representation of rapamycin-sensitive human LARP1 phosphorylated peptides, which were identified based on phosphorylation of conserved phosphorylation sites in LARP1 from *M. musculus (**47**). X axis* denotes residue number of human LARP1 (isoform 1019aa). *Y axis* denotes log2 area (DMSO/rapamycin). (**B**) Human LARP1 phosphorylation sites and corresponding tryptic phospho-peptides identified by mass spectrometry on LARP1 from *M. musculus* with annotations corresponding to human LARP1 isoforms 1019aa, 1096aa and 891aa. (**C**) Amino acid sequence surrounding for various human mTORC1 substrate. mTORC1-dependent phospho-residues are shown in boldface. (**D**) TOMTOM analysis of mTORC1-targets consensus sequence. Sequences from human S6K1, 4E-BP1, PRAS40 and Grb10 were used for this analysis. Elm2018 database was used for this analysis.

Having established that mTORC1 phosphorylates LARP1, we set out to comprehensively identify the rapamycin-sensitive residues. To this end, we mined publicly available mTORC1-sensitive phospho-proteome data (generated from rapamycin-treated TSC2^KO^ mouse embryonic fibroblasts (52)) for rapamycin-sensitive phospho-LARP1 peptides. These efforts culminated with the identification of 26 serine, threonine and three tyrosine rapamycin-sensitive phospho-residues in mouse LARP1—all of these phospho-residues are conserved in the human LARP1 protein (Figure 7A, B). Interestingly, from both functional and evolutionary standpoints, most of the rapamycin-sensitive phospho-serine and -threonine residues identified in our mouse screen are conserved across higher eukaryotes, from *D. rerio* to *H. sapiens* (Supplementary Figure S5). This is in contrast with the three rapamycin-sensitive phospho-tyrosines identified, which are not conserved in *D. rerio* (Supplementary Figure S5). Although there is precedent for tyrosine phosphorylation by mTOR (62,63), this kinase is primarily regarded as a serine/threonine kinase (30,37–39,42,43). For this reason, we focused our efforts on understanding the role of rapamycin-sensitive phosphorylation of serine and threonine residues on human LARP1.

We noted that in some instances, rapamycin impairs the phosphorylation of most of the rapamycin-sensitive residues identified, while in other cases it appears to enhance the phosphorylation of other residues (Figure 7A). Compare, for example, phosphorylated residues S247 and S689 within clusters 2 and 5, respectively: rapamycin treatment diminishes phosphorylation of some residues (log_2_ area [DMSO/rapamycin] value lies above the *x* axis) (e.g. S689); while, in other instances, rapamycin may enhance phosphorylation of others (e.g. S247, for which log_2_ area [DMSO/rapamycin] lies below the *x* axis) (Figure 7A, B). Rapamycin also affects phosphorylation of specific residues to different extents (Figure 7A, B); the phosphorylation of residues in cluster 6 is exquisitely sensitive to rapamycin, as denoted by the amplitude of the log_2_ area (DMSO/rapamycin) (Figure 7A, B), while rapamycin has minor effects on clusters 2 and 3. Collectively, these data indicate that rapamycin can alters LARP1 bi-directionally (inhibits the phosphorylation of some residues while enhancing that of others) and it does so to distinct stoichiometric levels (as denoted by the amplitude of the log_2_ area [DMSO/rapamycin] ratio).

### The amino acid sequences surrounding the rapamycin-sensitive phospho-residues in LARP1 resemble those of other mTORC1 targets, namely: 4E-BP1’s and PRAS40’s

Having identified the complete set of rapamycin-sensitive phospho-residues in LARP1, we set out to identify parallels between these phosphorylation events and those of other mTORC1 targets. mTORC1 directly catalyzes the phosphorylation of multiple serine and threonine residues within various proteins; these include: S6Ks (31,32,34), 4E-BPs (36–39,42,43), PRAS40 (47–49) and Grb10 (51,52) (Figure 7C,D). mTORC1 phosphorylates distinct target sequences. For example, T389 in S6Ks is found within the hydrophobic/aromatic motif (LG**F**T^389^**Y**VA, hydrophobic residues are underlined and aromatic residues shown in **boldface**) (Figure 7C). By contrast, the mTORC1 target residues (T37, T46, S65, T70, and S83) in 4E-BPs are invariably followed by proline at position +1, while the best-studied mTORC1-target site in PRAS40 (S183) is followed by a leucine at position +1 and a proline at position +2 (Figure 7C). Considerable divergence is observed between mTORC1 target sequences but a number of characteristics have been previously noted (54): mTORC1 shows a preference for a proline, hydrophobic (L,V) or aromatic residues (F,W,Y) at position +1 and a glycine at position –1. In addition, we note the preference for a methionine at +1 and a proline at +2 (Figure 7C). According to MEME analysis, the mTORC1 consensus target sequence can be loosely defined by the heptapeptide X-X-X-S/T-P/Y/L-V/P/R/G/D-X, where X denotes any amino acid (Figure 7D and reference (64)).

Interestingly, we observe that a number of rapamycin-sensitive phosphorylation sites in LARP1 align with the mTORC1 consensus sequence. For example, S440, S471, S689, S747 and S791 (numbering according to the 1019aa isoform 2 of LARP1) match the SxP sequence, characteristic of S183 in PRAS40 (47–49), while S444, T449, S697, S774, T779, T788 and T994 match the proline-directed sites in 4E-BPs (36–39,42,43) and within the auto-inhibitory loop of S6Ks. Lastly, S784 matches the hydrophobic/aromatic motif of S6Ks (31,32,34). Other sites (specifically, S770 and S979) are rapamycin-sensitive but follow the RxRxxS/T motif, characteristic of AGC kinases. These sites are therefore, in all likelihood, not direct targets for mTORC1, but rather targets for S6Ks (downstream effectors of mTORC1) *in vivo*. Hong and colleagues have previously shown that both S6K1 and AKT1 readily phosphorylate S770 and S979 *in vitro* (53).

### Phosphorylation of clusters 4 and 5 in LARP1 regulates its binding to mTORC1

**Figure 8.**
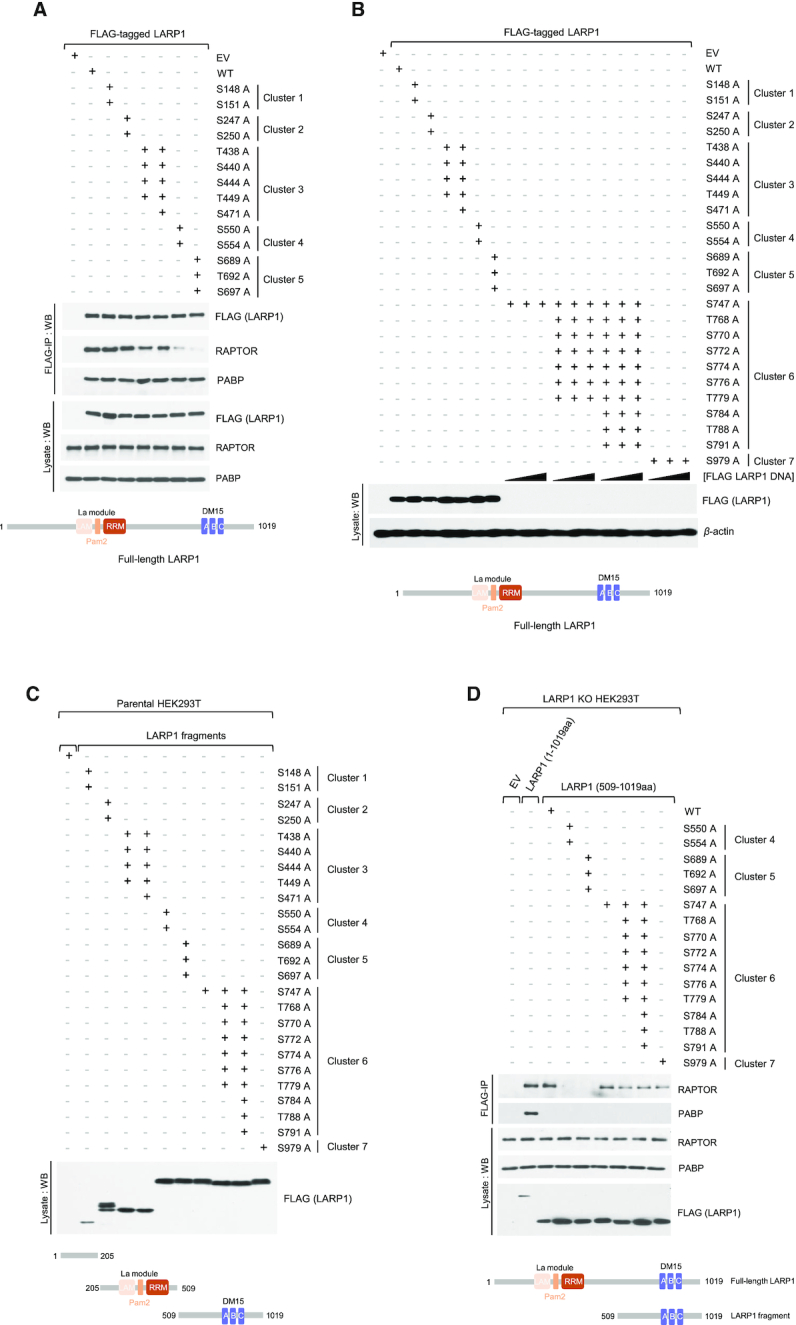
The C-terminal half of LARP1 interacts with RAPTOR dependent on Ser/Thr residues in clusters 4 and 5. (**A**) HEK293T cells were transiently transfected with empty vector (EV) or FLAG-tagged wildtype LARP1 or various phosphomutants of full-length LARP1. 24h after transfection, cells were harvested in CHAPS-based extraction buffer and samples were analyzed by SDS-PAGE/western blot using the indicated antibodies. Note that mutations within cluster 6 and 7 prevents expression of the LARP1 phosphomutants. (**B**) HEK293T cells were transiently transfected with the same vectors as in (A). Cells were stimulated with complete media for 3 h and then lysed in CHAPS-based extraction buffer. Lysates were subjected to immunoprecipitation with anti-FLAG antibody and were analyzed by SDS-PAGE/western blot using the indicated antibodies. (**C**) HEK293T cells were transiently transfected with empty vector (EV) or FLAG-tagged wildtype LARP1 or various phosphomutants of LARP1-fragments 1–205, 205–509 or 509–1019. Cells were then lysed and lysates were analyzed as in (A). (**D**) HEK293 LARP1 KO cells were transiently transfected with empty vector (EV), FLAG-tagged wildtype LARP1 or LARP1-fragment 509–1019 phosphomutants. Cells were treated and harvested and lysates were analyzed by immunoprecipitation and SDS-PAGE/western blotting as in (B).

We sought to understand the biochemical significance of LARP1 phosphorylation. Earlier, we mentioned that the interaction of LARP1 with mTORC1 is disrupted by rapamycin (Figure 5B). Similarly, depriving cells of amino acids reduces the association of LARP1 with mTORC1 (21), although not to the extent that rapamycin does. This raised the intriguing possibility that mTORC1 signalling-mediated phosphorylation of LARP1 regulates the interaction between LARP1 and mTORC1. To test this, we generated alanine-mutant versions of each rapamycin-sensitive phosphorylation site for each cluster individually and tested their interaction with RAPTOR in HEK 293T cells. Mutation of cluster 4 (comprising phospho-residues S550 and S554) and, especially, cluster 5 (comprising residues S689, T692, and S697) compromises the binding of ectopic LARP1 to endogenous RAPTOR but not its binding to PABP (Figure 8A). By contrast, mutation of clusters 1, 2 and 3 does not affect LARP1 association with RAPTOR. Mutation of phospho-residues (within clusters 6 and 7, flanking the DM15 motif) to alanine abrogates the steady-state expression of LARP1 (Figure 8B). These clusters could not, therefore, be tested for RAPTOR binding in the context of the full-length protein. To validate these results and overcome our inability to test the role of the C-terminal phosphorylation sites (clusters 6 and 7) on RAPTOR binding, we generated LARP1 fragments spanning the N-terminal region (amino acids 1–205), the mid-domain (amino acids 205–509), or the C-terminal region (amino acids 509–1019) bearing alanine in place of the identified rapamycin-sensitive phosphorylated residues (Figure 8C). These constructs were successfully transiently overexpressed in HEK 293T cells, thus overcoming the limitation of using full-length LARP1 constructs (*cf*. Figure 8B and C). We tested the interaction between endogenous RAPTOR with full-length wildtype LARP1 or that of a wildtype fragment of LARP1 spanning amino acids 509–1019, and equivalent fragments of LARP1 bearing alanine mutations in clusters 4, 5, 6 and 7. We transiently overexpressed these constructs in HEK 293T cells deleted for *LARP1* by CRISPR/Cas9 (Figure 8D). Use of the CRISPR/Cas9 LARP1^KO^ cells allowed us to avoid the competition between expressed constructs and endogenous LARP1 for RAPTOR binding; this is particularly important in light of our earlier observation that the wildtype 509–1019 fragment binds RAPTOR more weakly than full-length wildtype LARP1 does (Figure 3D). Consistent with the results obtained in Figure 8A, substitution of the rapamycin-sensitive phosphorylated-serine and -threonine residues in clusters 4 and 5 by non-phosphorylatable alanine residues impairs the binding of LARP1 to RAPTOR (Figure 8D). S689 and S697 in cluster 5 are particularly interesting because their surrounding sequences resemble those of other established mTORC1 targets (Figure 8D). The peptide sequence flanking S689 in LARP1 resembles that of S183 in PRAS40 (48,49), a rapamycin-sensitive direct mTORC1 phosphorylation site; while, S697 is followed by a proline, similar to the proline-directed mTORC1 sites in 4E-BP1: T37, T46, S65, T70 and S83 (42,43) (Figure 7C,D). Because S689, T692 and S697 were mutated to alanine as a group, we are unable to conclude which of these residues (if not all) is most important for the association of LARP1 with RAPTOR. We do know, however, that this effect is specific to clusters 4 and 5 because substitution of rapamycin-sensitive residues within clusters 6 and 7 do not affect the LARP1-RAPTOR interaction (Figure 8D). Collectively, these data suggest that phosphorylation of key serine/threonine residues within clusters 4 and 5 (and not in clusters 1, 2, 3, 6, and 7) regulate LARP1 binding to RAPTOR.

### mTORC1 signalling-mediated phosphorylation of LARP1 controls its TOP mRNA binding activity

**Figure 9.**
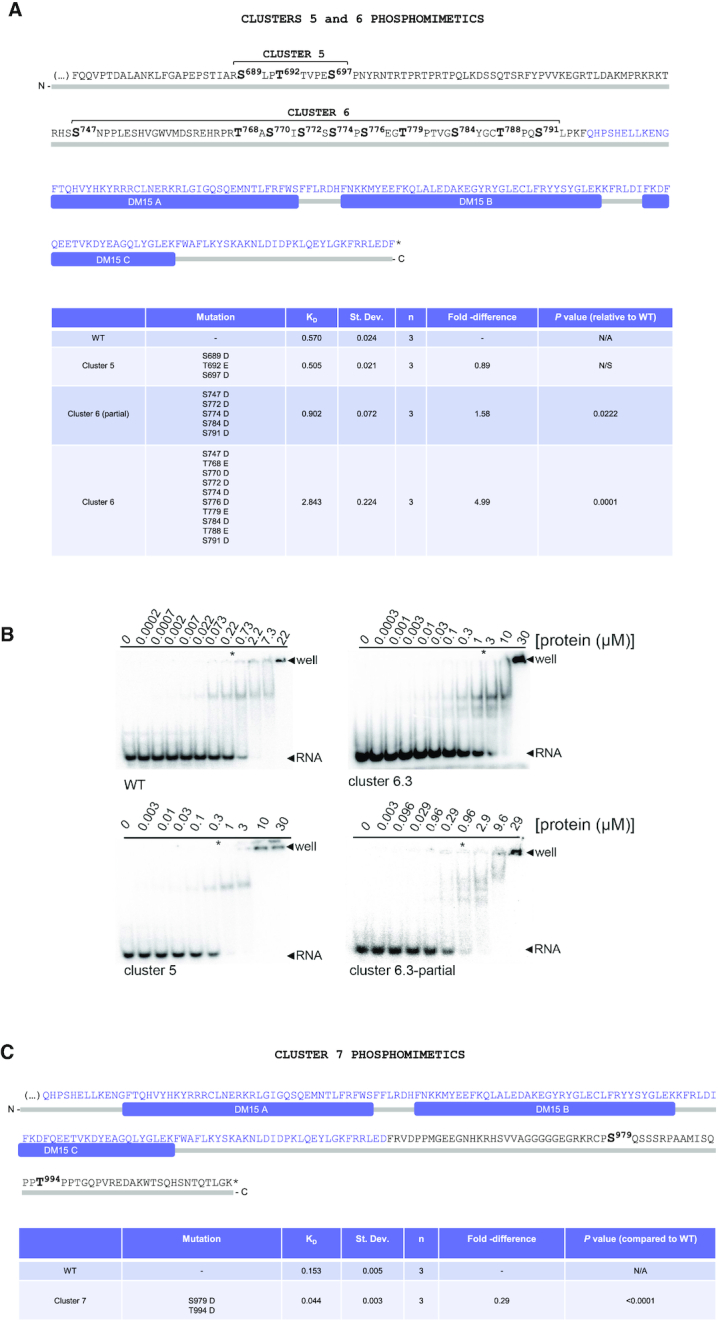
Phosphorylation of cluster 6 of LARP1 reduces its association with the 5′’UTR of RPS6 TOP mRNA. (**A**, **C**) Quantification of RNA-electrophoretic mobility shift assays for either WT or mutated versions of two distinct C-terminal fragments of LARP1 spanning residues 665–946 or 796–1019 (numbering according to LARP1 1019aa isoform). (**B**) Representative electrophoretic mobility shift assays of wildtype LARP1 construct (aa 665–964) with indicated phosphomimetic clusters. Radiolabeled capped RPS6 20-mer RNA oligo was incubated at 4°C with increasing concentrations of LARP1 protein and nonspecific competitors and resolved on a native gel. The asterisk indicates the average Kd of each construct from three independent replicates.

We next asked whether cluster 5 phosphoresidues could play a role in regulating the binding of LARP1 to the 5′UTR of TOP mRNAs. To answer this question, we bacterially-expressed a fragment of human LARP1 comprising an extended version of the DM15 motif (23,28) spanning residues (665–947) that encompasses S689, T692, and S697. Versions of this construct that contain a phosphomimetic aspartate or glutamate in place of these three residues were used to imitate the charge added by phosphorylation. We titrated each of these purified recombinant fragments into a radiolabeled RNA oligonucleotide containing the entire capped 5′UTR of RPS6 mRNA and analyzed binding affinity by electrophoretic mobility shift assay, as previously described (23). As shown in Figure 9A (table) and Figure 9B, the wildtype LARP1 fragment binds to m^7^G-RPS6-UTR with similar affinity (0.153 μM) to that of the S689D-T692E-S697D triple mutant, suggesting that phosphorylation of these residues may not be important for TOP mRNA binding. Therefore, we also asked whether the rapamycin-sensitive residues in cluster 6 (S747-T768-S770-S772-S774-S776-T779-S784-T788-S791) could play a role in TOP mRNA binding. Simultaneous mutation of each of these ten residues to a phosphomimetic aspartate or glutamate reduces the binding affinity of the LARP1 fragment for the 5′UTR of RPS6 by 4.99-fold (*K*_D_ = 2.843 μM) (Figure 9A, table Figure 9B). As expected, partial mutation of five selected phosphoresidues within cluster 6 (namely: S747-S772-S774-S784-S791) causes an intermediate decrease in LARP1 binding to the 5′UTR of RPS6 (*K*_D_ = 0.902 μM) (Figure 9A, table and Figure 9B). By contrast, substitution of two phosphoresidues in cluster 7 (S979 and T994) for an aspartate and glutamate sufficed to enhance the binding of LARP1 fragment (796–1019) to the 5′UTR of RPS6 (*K*_D_ = 0.044 μM) (Figure 9C, table). Phosphomimetic mutations do not always recapitulate phosphorylation events, nevertheless these data suggest that mTORC1-mediated phosphorylation of LARP1 at clusters 6 and 7 regulates the RNA-binding activity of LARP1 in an opposing fashion.

### mTORC1, not S6K1, regulates the binding of LARP1 to TOP mRNAs

**Figure 10.**
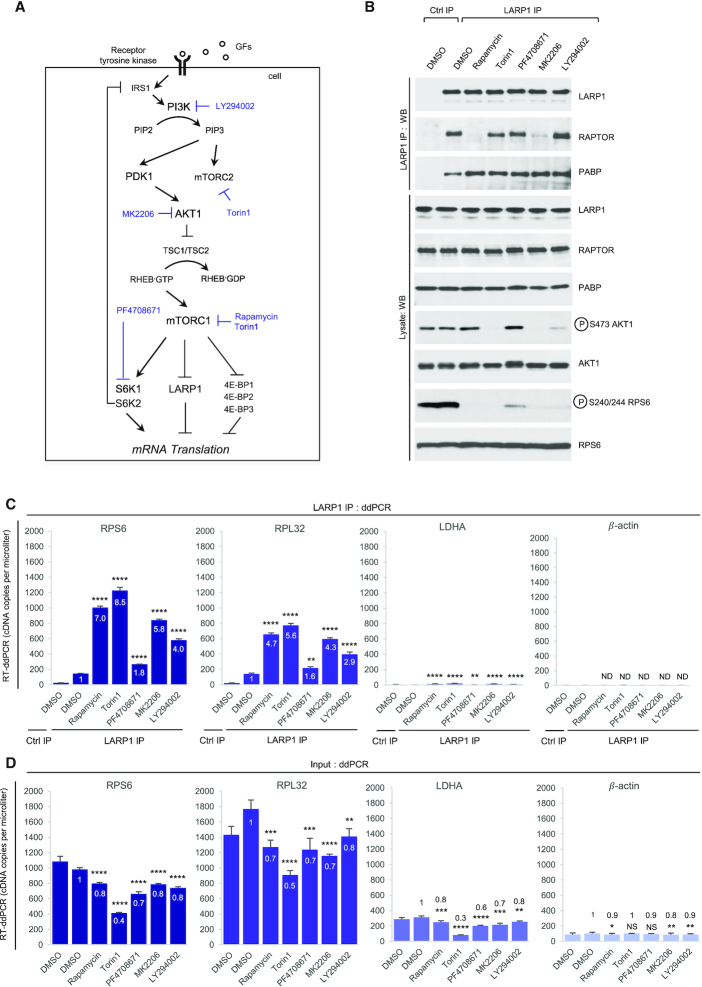
Chemical inhibition of the PI3K/AKT1/mTORC1 pathway enhances the binding of endogenous LARP1 to TOP and non-TOP mRNAs. (**A**) Diagram depicting the PI3K/AKT1/mTORC1 signalling pathway and pharmacological agents targeting specific kinases within this pathway. (**B**–**E**) HEK293T cells were stimulated with complete media for 3 h in the presence of DMSO (vehicle) or the indicated drugs (see *Experimental Procedures* section for concentration details). Cells were lysed in CHAPS-based extraction buffer in the absence of RNase A and lysates were subjected to protein analysis by SDS-PAGE/Western blot using the indicated antibodies (B) or used for RNA co-immunoprecipitation using anti-LARP1 antibodies and analyzing RNA pull-downs by RT-ddPCR (**C**, **D**). *Numbers* within or above bars denote fold enrichment over DMSO/LARP1 IP sample. Asterisks reflect statistical significance: * denotes *P*-value < 0.05, ** denotes *P*-value < 0.01, *** denotes *P*-value < 0.001 and **** denotes *P*< 0.0001. NS signifies non-significant. ND signifies non-determinable.

The PI3K/AKT1/TSC/RHEB/mTORC1 pathway is widely recognized to play a fundamental role in the control of TOP mRNA translation (14,17,18,18,19,19,20,65). To dissect the contribution of each component of the PI3K/AKT1/TSC/RHEB/mTORC1 pathway and its downstream substrate S6K1 (see Figure 10A) to the binding of LARP1 to TOP mRNAs, HEK 293T cells were acutely treated with mTOR inhibitors (rapamycin or torin1), an S6K1 inhibitor (PF4708671 (66)), an allosteric pan-AKT inhibitor (MK2206 (67)) or a low-specificity PI3K inhibitor (LY294002 (68,69)) and lysates subjected to protein immunoprecipitation (Figure 10B) and RNA immunoprecipitation (RNA-IP) (Figure 10C) using an antibody against endogenous LARP1. The resulting data show that endogenous LARP1 preferentially interacts with highly abundant TOP mRNAs (*e.g*. RPS6 and RPL32, tested here) compared to other less abundant non-TOP mRNAs (e.g. lactate dehydrogenase A (LDHA) and beta-actin (β-actin) (Figure 10C, Supplemental Figure S2A), even upon normalization of LARP1-bound mRNA levels to their total abundance in the lysate/input (see Statistical Workbook). Drug treatment has modest, but statistically significant, effects on mRNA steady-state levels. Torin1 has the most drastic effect on mRNA steady-state levels; HEK 293T cells treated with 300 nM torin1 for as short as 3 h have significantly (*P* < 0.0001) decreased mRNA RPS6, RPL32 and LDHA mRNA association with LARP1 (Figure 10D); this was not evident for β-actin mRNA (*P*-value is non-significant) possibly due to its intrinsic low abundance compared to the other mRNAs. Importantly, incubation of cells with rapamycin, torin1, MK2206, and LY294002 enhances the binding of LARP1 to all mRNAs tested including TOP (RPS6 and RPL32) and non-TOP (LDHA and β-actin) mRNAs (Figure 10C). Rapamycin leads to a 7-fold enrichment of RPS6 mRNA binding to LARP1 and a 4.7-fold enrichment of RPL32 mRNA (Figure 10C). Similar fold-changes are observed when LARP1 immunoprecipitation data is normalized to steady-state input mRNA levels (see Statistical Workbook). As observed for rapamycin, torin1 also significantly (*P* < 0.001) enhances the association of both TOP and non-TOP mRNAs with LARP1 (Figure 10C). Torin1 appears to enhance mRNA association with LARP1 more efficiently than rapamycin does, consistent with it inhibiting mTORC1 more strongly than rapamycin does; this is particularly evident when LARP1 IP data is normalized to steady-state mRNA input levels: for example, rapamycin induces an 8.5-fold enrichment of RPS6 mRNA, while torin1 leads to a 20.1-fold enrichment of the same transcript in LARP1 IPs (see Statistical Workbook in Supplemental Data). A similar pattern was observed for the remaining mRNAs tested. It appears, therefore, as though torin1 is more effective than rapamycin is at enhancing the binding of LARP1 to mRNA. The allosteric pan-AKT inhibitor (MK2206) and the non-specific PI3K inhibitor (LY294002) also enhanced the binding of TOP and non-TOP mRNAs to LARP1 protein, albeit less efficiently than does rapamycin or torin1 (Figure 10C). This is consistent with the fact that PI3K/AKT1 function upstream of mTORC1.

It was previously shown that LARP1 associates with mTORC1 (21), and while both rapamycin and torin1 enhance the association of LARP1 with TOP and non-TOP mRNAs, it is unclear whether mTORC1 does so directly or indirectly through a downstream kinase. Ribosomal protein S6 kinases (S6K1 and S6K2) are positioned downstream of mTORC1, and S6K1 has been shown to phosphorylate LARP1 *in vitro* (53). To determine whether S6K1 plays a role in the binding of LARP1 to TOP and non-TOP mRNAs in cells, we monitored the effects of the specific S6K1 inhibitor PF4708671 (66) on the binding of endogenous LARP1 to TOP and non-TOP mRNAs. In contrast to rapamycin and torin1, PF4708671 has only a minor effect on LARP1 binding to TOP and non-TOP mRNAs (Figure 10C). PF4708671 treatment leads to rather small (1.8-fold and 1.6-fold), and in some instances statistically insignificant, increases in LARP1 binding to RPS6 and RPL32 mRNAs, respectively (Figure 10C). This observation is consistent with other studies that exclude S6Ks from playing a role in TOP mRNA translation (8,14,18,70).

Since rapamycin (but not torin1) impairs the binding of LARP1 to mTORC1 (Figure 5B), we also performed protein LARP1 IPs and monitored the effect of these drugs on the interactions between endogenous LARP1 and RAPTOR. As observed in Figure 5B, treating cells with rapamycin disrupts the interaction between LARP1 and RAPTOR while torin1 has only a negligible effect on the interaction between these proteins (Figure 10B). Neither the S6K1 inhibitor (PF4708671) nor the low-specificity PI3K inhibitor (LY294002) reduce the association of LARP1 with RAPTOR (Figure 10B). By contrast, the allosteric pan-AKT inhibitor (MK2206) decreases the interaction between LARP1 and RAPTOR, but not that of LARP1 with PABP. That rapamycin, torin1, MK2206, and LY294002 increase the binding of LARP1 to TOP mRNAs (Figure 10C) but only two of these drugs (rapamycin and MK2206) visibly impairs LARP1-RAPTOR binding, suggests that dissociation of LARP1 from RAPTOR is not required for LARP1 to bind TOP mRNAs.

### mTORC1-mediated phosphorylation of LARP1 regulates its TOP mRNA translation inhibitory activity

**Figure 11.**
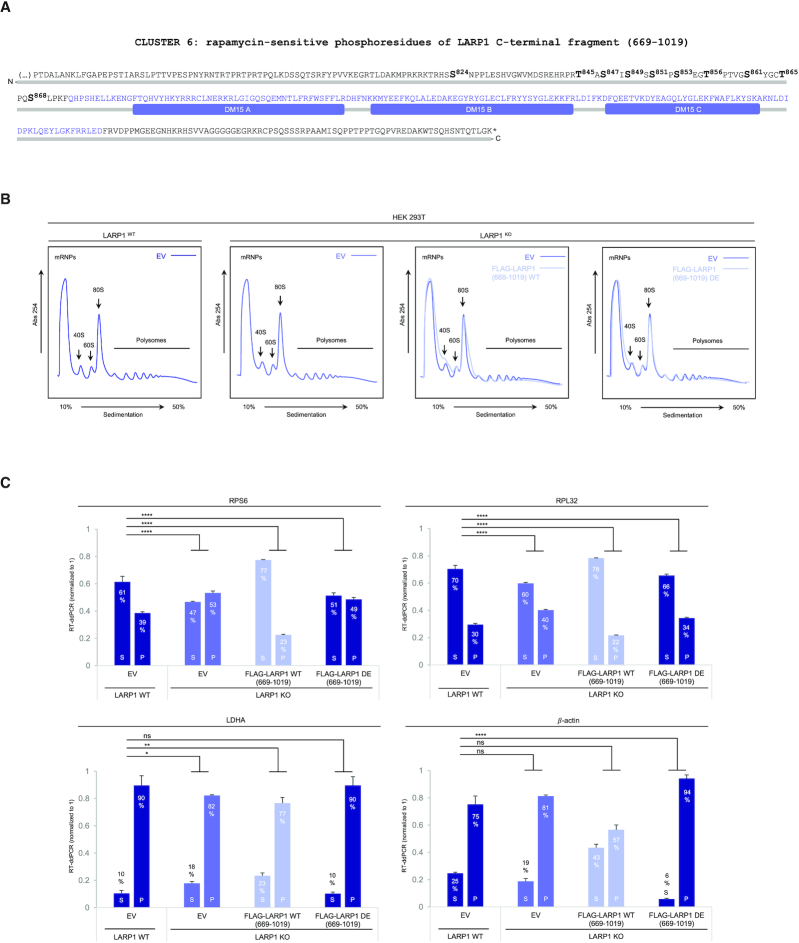
Phosphorylation of cluster 6 in LARP1 C-terminal fragment of LARP1 (spanning residues 669–1019) derepresses TOP mRNA translation. (**A**) Amino acids sequence and diagrammatic representation of the C-terminal fragment of human LARP1 comprising the DM15 domain proximal context and key rapamycin-sensitive cluster 6 phosphorylation sites, which were mutated to generate LARP1 DE (669–1019). Although amino acid numbering is shown according to 1019 isoform, this segment of the protein shows 100% identity at the amino acid level between all known human LARP1 isoforms. (**B**) HEK293T LARP1 WT and LARP1 KO cells were cultured to near-confluency (∼70–80%) at which point cells were replenished with fresh complete media (containing 10% (v/v) fetal bovine serum) for 4 h. Cells were lysed in hypotonic buffer and samples of lysates were subjected to sucrose gradient ultracentrifugation/polysome profile analysis. Absorbance (254 nm) profiles are shown. The same EV profile for LARP1 KO cells is shown in every one of the three LARP1 KO panels, strictly for profile comparison purposes. (**C**) Total RNA was extracted from each fraction and pooled into subpolysomal (S) and polysomal (P) fractions. cDNA was synthesized from S and P fractions by reverse-transcription (RT) and specific transcripts quantified by droplet digital PCR (ddPCR). Data shown as percentage abundance on S and P fractions. *Error bars* denote standard deviation (St. Dev.) for three technical replicates.

Having identified the key mTORC1-dependent phosphorylation sites on LARP1 that regulate its ability to interact with TOP mRNA, we sought to investigate whether mTORC1-mediated phosphorylation of LARP1 de-represses TOP mRNA translation. LARP1^WT^ cells were transiently transfected with empty vector (EV), while LARP1^KO^ cells were transiently transfected with EV, FLAG-LARP1 C-terminal fragment (669–1019) wildtype (WT) or an identical fragment that contains aspartate or glutamate mutations in place of the phospho-serine and -threonine residues in cluster 6 (Figure 11A); cluster 6 mutants were selected because they display the strongest effect on TOP mRNA binding (Figure 9A). Transfected cells were subjected to polysome profiling analysis (Figure 11B). While overexpression of the wildtype or the phosphomimetic LARP1 fragments does not noticeably alter the polysomal profiling traces (Figure 11B), it alters TOP mRNA distribution (Figure 11C); as observed in earlier profiles (Figure 1B,C), LARP1^KO^ cells exhibit higher levels of RPS6 and RPL32 transcripts in the polysome (P) fraction than wildtype cells do. This is evident also in Figure 11C with 53% of total RPS6 transcript found in the (P) fraction in LARP1^KO^ cells compared to 39% in LARP1^WT^ cells. The RPL32 transcript is also more abundant in the (P) fraction of LARP1^KO^ cells than the LARP1^WT^ counterpart, with 40% of the total amount found in the (P) fraction in LARP1^KO^ compared to 30% in LARP1^WT^ cells. Transient overexpression of the wildtype C-terminal fragment of LARP1 (residues 669–1019) re-establishes translation repression of RPS6 and RPL32 mRNAs to the levels observed for wildtype cells. In contrast, the equivalent LARP1 C-terminal fragment that bears phospho-mimetic mutations fails to repress RPS6 and RPL32 translation (Figure 11C). Importantly, these data demonstrate that mTORC1-mediated phosphorylation of key serine and threonine residues within cluster 6 causes de-repression of TOP mRNA translation. mTORC1, therefore, controls TOP mRNA translation *via* the modification of specific residues of LARP1.

## DISCUSSION

The PI3K/AKT1/TSC/RHEB/mTORC1 pathway is widely recognized to play a fundamental role in the control of TOP mRNA translation (14,17,18,18,19,19,20,65), but the key effector molecule downstream of this pathway that regulates this process remained a mystery for many decades (8). Early observations that rapamycin simultaneously blocks S6K phosphorylation (31,32) and impairs TOP mRNA translation (13,15) led some (71) to postulate that mTORC1 controls TOP mRNA translation *via* S6Ks. This early model – based solely on correlative analyses – was later disproved by causal experimentation (14,18,72); most notably, it was observed that genetic deletion of *S6K1* and/or *S6K2* does not alter the sensitivity of TOP mRNAs to the inhibitory effects of rapamycin on the translation of these transcripts (14,18,72). In the years that followed, a number of other proteins and regulatory RNAs were also suggested to regulate TOP mRNA translation (reviewed in (1)) but none of them withstood the rigorous test of time (8).

**Figure 12.**
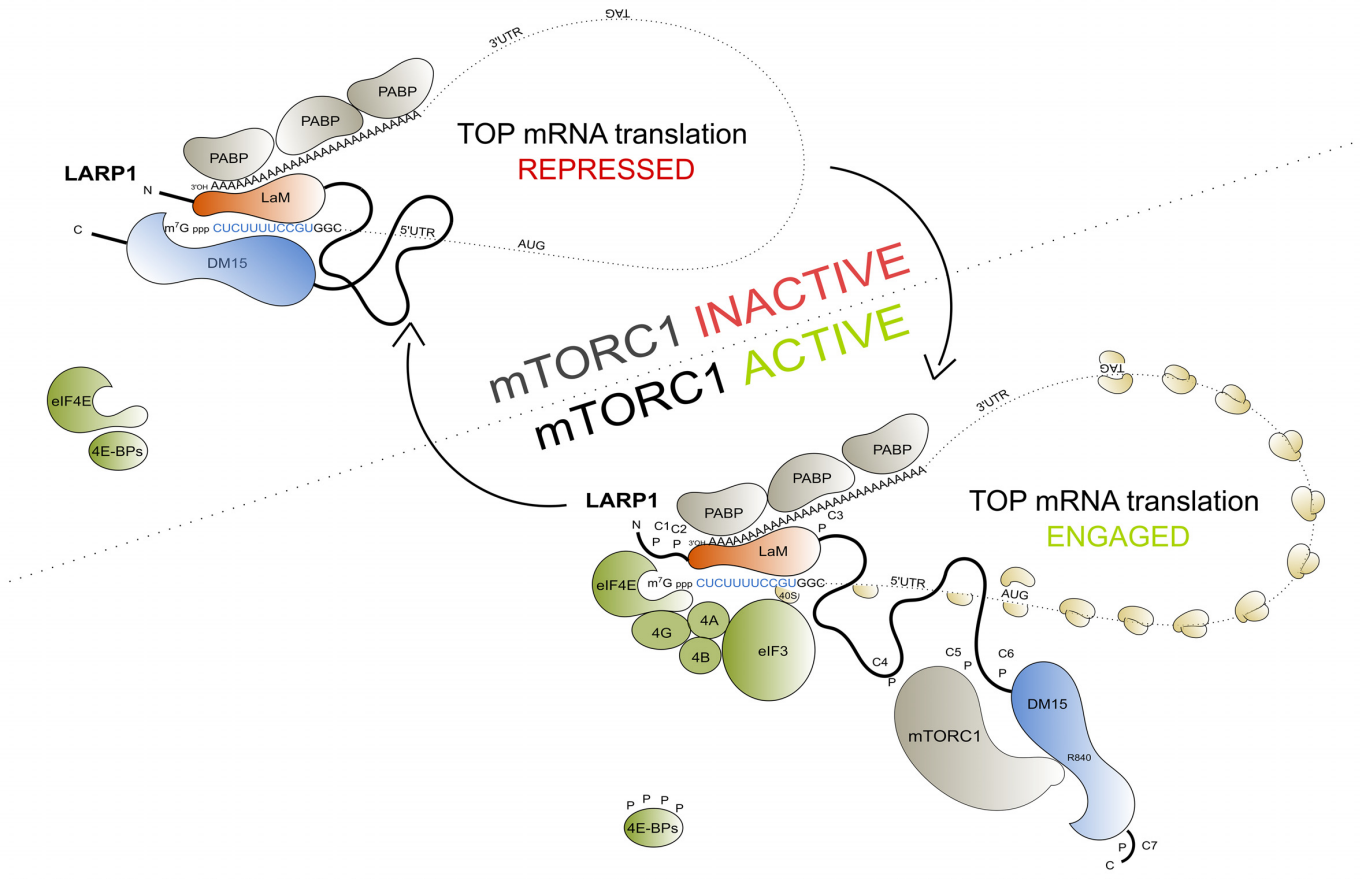
Diagram depicting putative model for mTORC1-mediated release of LARP1 for the N^7^-methyl guanosine triphosphate (m^7^Gppp) cap structure and adjacent 5′ terminal oligopyrimidine (5′TOP) motif. mTORC1 phosphorylates multiple serine and threonine residues. Phosphorylation of cluster 6, in particular, contributes to the release of LARP1 from the 5′UTR of RPS6 mRNA, Thus allowing for the assembly of the eIF4F complex on 5′TOP mRNAs. Concurrently, mTORC1 phosphorylates multiple serine and threonine residues on 4E-BPs, releasing the latter protein from eIF4E and allowing for the association of eIF4E with eIF4G which together with eIF4A and eIF3 recruit the 40S subunit of the ribosome to TOP mRNAs for translation initiation.

Recent data (21,24) suggest that LARP1 functions as the elusive repressor of TOP mRNA translation downstream of mTORC1 (reviewed in (22)). The mTORC1-LARP1 signalling axis was originally discovered independently by two groups a few years ago now (21,26). Tcherkezian et al. postulated that LARP1 positively regulates the translation of TOP transcripts through direct interactions with eIF4E and PABP (26). In contrast, we (21) observed that, while LARP1 indeed binds PABP as originally reported (73,74), it does not bind eIF4E nor does it bind eIF4G1 (21). Notably, we also concluded, through multiple experimental lines of evidence, that LARP1 functions, in fact, as a repressor, not as an activator, of TOP mRNA translation (21); the repressor model has since been corroborated by others (24). In this context, the DM15 domain has been recently shown to play a key role in TOP mRNA translation repression (24). LARP1 (*via* its DM15 region) binds the m^7^Gppp cap and adjacent 5′TOP motif of TOP mRNAs, thus blocking access of eIF4E to the cap and the assembly of the eIF4F complex on the 5′UTR of TOP transcripts (23). An extended version of the DM15 has been shown to repress the translation of TOP mRNAs (24). LARP1 effectively inhibits cap-dependent TOP mRNA translation in living cells (21,24) and *in vitro* (24). Notably, depletion of LARP1 by RNAi (21) or genetic deletion (24) renders cells partially resistant to the inhibitory effects of rapamycin and torin1 on TOP mRNA translation, indicating that LARP1 functions as a translation repressor of TOP mRNA translation downstream of mTORC1. The present study substantiates these early findings (that LARP1 represses TOP mRNA translation *via* its DM15 region, Figures 1 and 2) and reveals new details that significantly expand our molecular understanding of how mTORC1 controls this process (Figures 3 to 11). Collectively, these new findings lead us to propose an improved model of LARP1-mediated repression of TOP mRNA translation depicted in Figure 12.

Two important proteome-wide phosphoscreens, which aimed to identify novel signalling pathways downstream of mTORC1 (51,52), were the very first studies to document the effects of rapamycin on LARP1 phosphorylation. A follow up study by Kang *et al.* (54) aimed at understanding mTORC1 substrate-specificity and rapamycin-sensitivity subsequently revealed that mTORC1 can directly catalyze the phosphorylation of two residues on LARP1 *in vitro*: S766 and S774 (amino acid numbering according to the 1096 amino acid-long isoform—the corresponding numbering on the 1019 aa-long isoform used in our study and hereafter for simplicity are S689 and S697). Phosphorylation of S689 and S697 is equally sensitive to torin1, but exhibited differential sensitivity to rapamycin: S689 is reportedly largely resistant while S697 is sensitive to this drug (54). This conclusion is somewhat surprising because the sequence flanking S689 resembles that of S183 on PRAS40 (a rapamycin sensitive site (48,49)) while S697 is a proline-directed site identical to those found in 4E-BP1 (Figure 8), whose phosphorylation is largely rapamycin resistant (Diggle *et al.*, 1996). Hong *et al.* (53) confirmed that LARP1 is, indeed, a direct substrate of mTOR *in vitro*. Using a GST-LARP1 fragment spanning residues 654–731 bearing a double mutation on S689A/T692A, they show that mTOR-mediated phosphorylation is lost. These data suggest that S689 and/or T692 are the primary mTOR phosphorylation sites on this fragment of LARP1 (53). To begin addressing the mechanism by which mTORC1 controls LARP1 function, we performed *in vivo* orthophosphate labeling experiments in the presence of mTOR inhibitors and complemented these findings with mTORC1 *in vitro* kinase assays against LARP1 (Figure 6); we have also identified 26 rapamycin-sensitive phosphorylation sites on LARP1: S148, S151, S247, S250, T438, S440, S444, T449, S471, *S550*, *S554*, *S689*, *T692*, *S697*, S747, T768, S770, S772, S774, S776, T779, S784, T788, S791, S979 and T994 (numbering according to the 1019 aa isoform) (Figure 7) that are conserved across the animal kingdom (Supplementary Figure S5). Phospho-residues in clusters 4 and 5 (shown in *italics*) are important for the LARP1 association with the mTORC1 complex (Figure 8), while underlined phosphoresidues in cluster 6 are important for its association with the 5′UTR of RPS6 (TOP) mRNA (Figure 9). The phosphoresidues in cluster 6 are also determinant for LARP1-mediated repression of TOP mRNA translation (Figure 11). Importantly, although phosphoresidues S689 and S692 (within cluster 5) are, indeed, phosphorylated in a mTORC1-dependent manner as reported by (53), these residues do not appear to regulate TOP mRNA binding (Figure 9). Cluster 6, not cluster 5, comprises the key phosphoresidues that control LARP1’s RNA binding and translation inhibitory activities (Figure 9). Additional work will be required to gain a full understanding of the precise molecular consequences of LARP1 phosphorylation. Our study provides the first piece of direct evidence for a role for mTORC1-mediated LARP1 phosphorylation on TOP mRNA binding and translation control.

LARP1 belongs to the LARP superfamily of proteins that comprises seven members: LARP1, LARP2 (also known as LARP1B), La (sometimes referred to as LARP3), LARP4, LARP5 (also known as LARP4B), LARP6 and LARP7. LARP2 is the closest homolog of LARP1 (with 60% amino acid sequence identity and 73% amino acid sequence similarity) (Supplementary Figure S3). Despite their high degree of sequence conservation, here we show that while both LARP1 and LARP2 bind PABP (through a conserved La module) only LARP1 is capable of strongly interacting with mTORC1 (Figure 3). In our hands, myc/Flag-LARP2 interacts rather weakly with mTORC1. The interaction between mTORC1 and LARP2 can, for all accounts and purposes, be considered negligible (Figure 3). The recent report by Hong and colleagues (53) proposed that myc-LARP2 interacted strongly with Flag-mTOR/HA-RAPTOR; however, our data suggest that their finding is likely a non-specific effect resultant from simultaneous overexpression of mTOR, RAPTOR and LARP2. Interestingly, although in our hands LARP2 interacts weakly with endogenous mTORC1 (and is therefore an unlikely physiological mTORC1 target), many of the serine/threonine rapamycin-sensitive phosphorylation sites on LARP1 are also conserved in LARP2 (e.g. every serine/threonine phospho-residue in clusters 2 through 5 is conserved in LARP2) (Supplementary Figure S3), thus raising the intriguing possibility that LARP2 is also a phosphoprotein. Similarly, all cluster 6 residues except T779 of LARP1 are also conserved in LARP2, where LARP2 has an alanine in that position. Critically, some of the conserved LARP1/LARP2 phospho-residues show conservative serine to threonine or threonine to serine substitutions between LARP1 and LARP2; for instance, T449 in cluster 3 of LARP1 is a serine in LARP2 (Supplementary Figure S3). Conservative substitutions suggest that, despite sequence divergence between these two closely related proteins, there was evolutionary pressure to retain phosphorylation. The identity of the kinase(s) that phosphorylates LARP2 is unknown at present, as is the physiological significance of LARP2 phosphorylation. Considerable additional work is required to elucidate the molecular function(s) of LARP2 and its mechanism of regulation.

The present manuscript focuses on the study of the physical and functional interactions between mTORC1 and LARP1. In addition to interacting with mTORC1, LARP1 also interacts with PABP (21,26,60,73). But how does LARP1 interact with PABP and what is its physiological significance? LARP1 employs distinct regions to bind PABP and mTORC1 (Figure 3; see also Figure 12 for schematic representation). In the present study, we show that LARP1 binds to PABP *via* the mid-domain region (spanning amino acids 205–509) that comprises the La module (formed by 3 motifs: the La motif, the PAM2 motif and the RNA-recognition motif) (Figure 3). This finding is consistent with our earlier observation that the PAM2 motif situated between the La motif and the RRM_L5_ is essential for the interaction of LARP1 with PABP (21). The PAM2 motif is conserved in LARP2; perhaps not surprisingly, LARP2 interacts with PABP to a similar extent as LARP1 (Figure 3). The significance of the LARP2 interaction with PABP remains undefined. Other LARP superfamily proteins LARP4 (56) and LARP5 (75) also interact with PABP. They do so through two distinct regions: (1) *via* an atypical PAM2w motif located in the N-terminal region of LARP4 and LARP5 (56) and (2) *via* a PABP-binding motif (PBM) positioned C-terminally of the RRM_L4_ (75). LARP1 also interacts with the poly(A) tail (60) *via* the LaMod (76). The physiological significance of the interaction of LARP1 with PABP and the poly(A) tail remains incompletely defined. LARP1 has been previously shown to regulate the stability of TOP transcripts (21,60,77). A recent study by (78) demonstrated that LARP1 exhibits poly(A) tail lengthening activity – it is therefore tempting to speculate that may LARP1 bind to PABP and the poly(A) tail of TOP mRNAs to protect these transcripts from deadenylation.

While the domains by which LARP1 interacts with PABP are well-defined, the interaction between LARP1 and mTORC1 is poorly understood. The data reported in the present study reveal that the C-terminal region spanning residues 509–1019 (that comprises the DM15 region and some adjacent sequences) is important for the interaction of LARP1 with RAPTOR (and by extension mTORC1) (Figure 3). These findings are consistent with a recent study by Thoreen and colleagues (24) in which the authors report that amino acids 497–1019 on LARP1 mediate RAPTOR binding. The question remains as to which specific residues in LARP1 are required for binding to RAPTOR. We show that R840 (within the DM15 region) is of primary importance in this context, but multiple contact points in various regions of LARP1 are likely required for RAPTOR binding, evidenced by our observation that the C-terminal fragment of LARP1 interacts rather weakly with RAPTOR, when compared to the full-length protein (Figure 3). It is also possible that proper LARP1 folding, feasible only in the context of the full-length protein, is required for RAPTOR association.

### The ‘Pendular Hook’ translation repression model

Previously, we showed that LARP1 interacts with PABP *via* a PAM2 motif nudged between the La motif and the RRM_L5_ and that rapamycin does not regulate the interaction with PABP (21). Consistent with these findings, herein we report that, although rapamycin inhibits the phosphorylation of residues within the RRM_L5_ in cluster 3 (T438, S440, S444, T449 and S471) (Figure 7), mTORC1 does not alter the binding of the LARP1 LaMod to PABP (21) (Figure 5B); it could, however, hypothetically regulate the binding of the La module to the poly (A) tail or TOP sequence (76). By contrast, mTORC1-mediated phosphorylation of cluster 6 releases the DM15 region from the 5′UTR of RPS6 (Figure 9A), thus allowing for TOP mRNA translation to take place (Figure 11A-C). The observation that mTORC1 regulates the binding of the DM15 region to the 5′TOP motif, but does not affect the LaMod-PABP interaction, leads us to propose a refined model for LARP1-mediated translation repression, in which the N-terminal region of LARP1 remains constitutively bound to PABP, while the DM15 region is poised and ‘hooks’ (i.e. engages) the m^7^Gppp cap and 5′TOP motif binding only in conditions of mTORC1 inactivation. We hypothesize that mTORC1 activation, and consequential DM15-proximal phosphorylation of cluster 6, ‘unhooks’ (i.e. releases) the DM15 module in a ‘pendular’ motion because the La module remains associated with PABP (21). We propose a novel LARP1 translation repression model in which mTORC1 coordinates the ‘pendular hook’-motion of LARP1 (Figure 12). How does this conformational change come about? The sequence of phosphorylation events that may lead to this conformational change is not known; the available evidence suggests that clusters 4 and 5 may play a role in mediating this conformational change in that they appear to be required for ‘docking’ of mTORC1 onto LARP1. The present study delineates a function for LARP1 phosphorylation in RNA-binding activity and translation control. This study serves as a primer for further detailed study of TOP mRNA translation control by LARP1. Careful biochemical and structural analysis of this novel signalling pathway will likely yield invaluable insights into the control of TOP mRNA translation and, by extension, ribosome biogenesis—a fundamental basic cellular process. Such findings will ultimately help unravel novel therapeutic opportunities for the treatment of diseases characterized by dysregulated mTORC1-LARP1 signalling and ribosome biogenesis. Future structural work will certainly help delineate in atomic detail the precise mechanism by which mTORC1 engenders LARP1 inactivation.

## Supplementary Material

gkaa1239_Supplemental_Files

## Notes

Present address: Bruno D. Fonseca, PrimerGen Ltd., Viseu, Portugal.

Present address: Marius R. Niklaus, Institute of Clinical Transfusion Medicine and Haemotherapy, University of Wuerzburg, Wuerzburg, Germany.

Present address: Izabella A. Pena, Whitehead Institute for Biomedical Research, Department of Biology, Massachusetts Institute of Technology (MIT), Cambridge, USA.

Present address: Mark Livingstone, Livingstone Conseil SASU, Versailles, France.
