# Supplementary material for: mTORC1 promotes TOP mRNA translation through site-specific phosphorylation of LARP1
Source: Nucleic Acids Res. 2021 Jan 4;49(6):3461–89. doi: 10.1093/nar/gkaa1239 (PMC8034618; doi:10.1093/nar/gkaa1239)
Supplement: gkaa1239_Supplemental_Files [file gkaa1239_supplemental_files.zip › 2020-08-08 Suppl. Figures (merged).pdf]

A

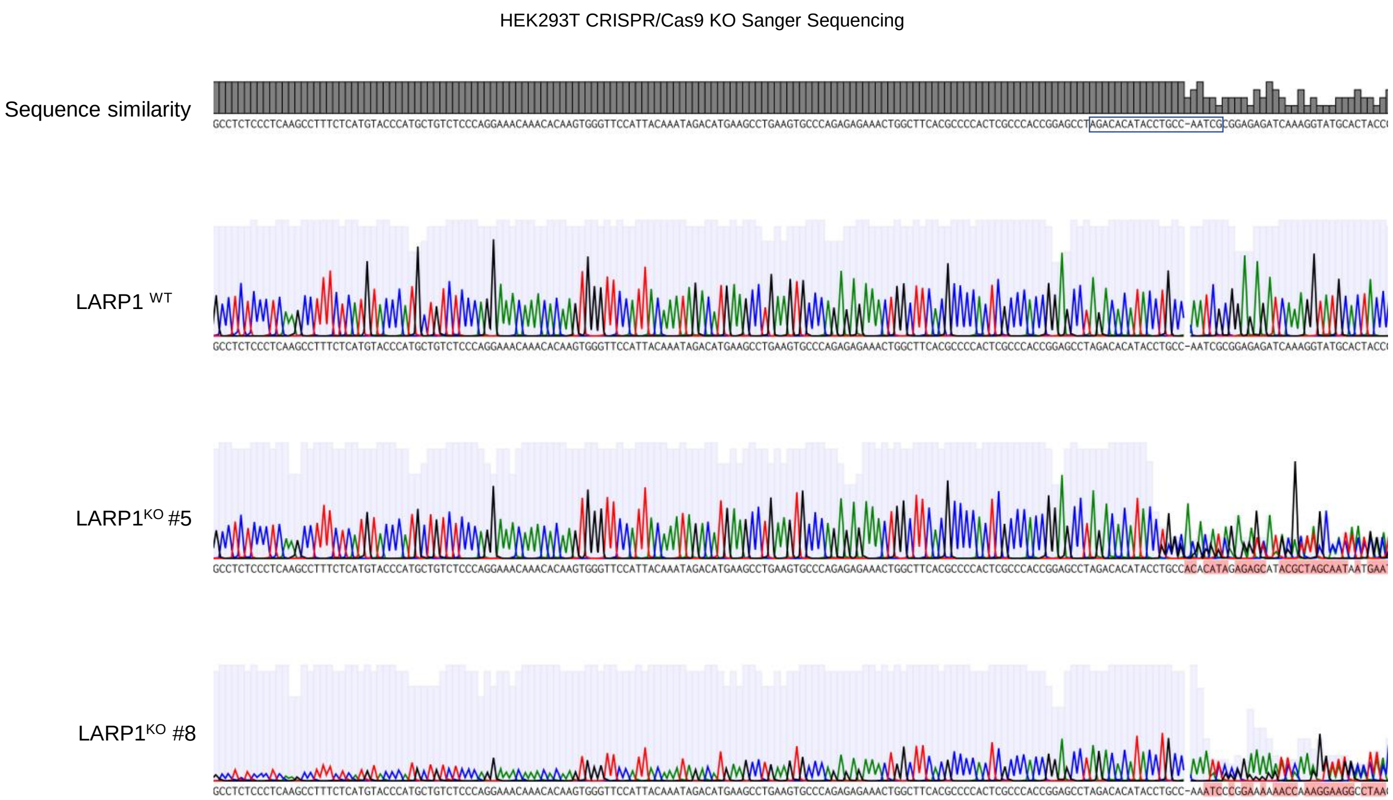

C

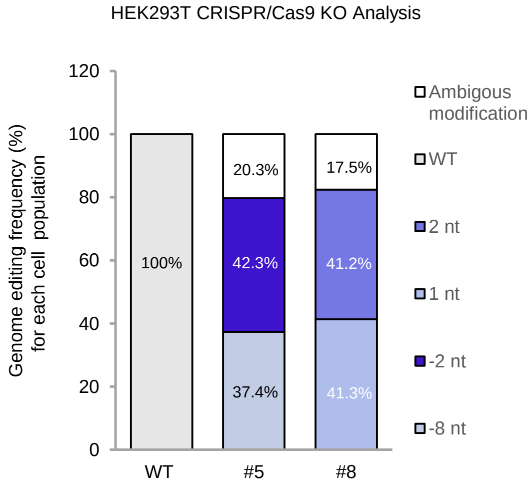

D

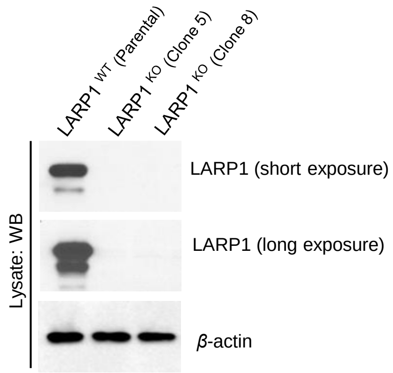

B

HEK293T CRISPR/Cas9 KO Genomic Sequence

Clone		Genomic Sequence
LARP1 WT		5'-TAGACACATACCTGCCAATCGCGGAGAGATCAAA-3'
LARP1 KO #5	Allele 1	5'-TAGACACATACCTGCCA* *CGCGGAGAGATCAAA-3'
	Allele 2	5'-TAGACACATACC* * * * * * * *GCGGAGAGATCAAA-3'
LARP1 KO #8	Allele 1	5'-TAGACACATACCTGCCAA**A**TCGCGGAGAGATCAAA-3'
	Allele 2	5'-TAGACACATACCTGCCAA**AA**TCGCGGAGAGATCAAA-3'

Suppl. Figure 1. Genomic editing of human *LARP1* gene locus by CRISPR/Cas9. (A) Sanger sequencing chromatograms of PCR products spanning the sgRNA targeting site (boxed sequence) from unedited (LARP1 WT) and edited clones. (B) Genomic sequencing results for the LARP1 locus near the targeted site of the LARP1 gene. Each * marks deletion of a nucleotide and bold with underlining marks insertion. (C) Frequency (%) of genome editing events within *LARP1* genomic region for each of the CRISPR/Cas9 cell clones determined by TIDE analysis of Sanger sequencing chromatograms (shown in A). *2nt* denotes 2 nucleotide insertion; *1nt* denotes 1 nucleotide insertion; *-2nt* denotes 2 nucleotide deletion; *-8nt* denotes 8 nucleotide deletion; Sanger sequencing reads that suggested genomic alterations but lack statistical power are shown as *ambiguous modifications*. (D) Western blot analysis of LARP1 expression in LARP1 WT and LARP KO clonal cell lines.

Suppl. Figure 2

A

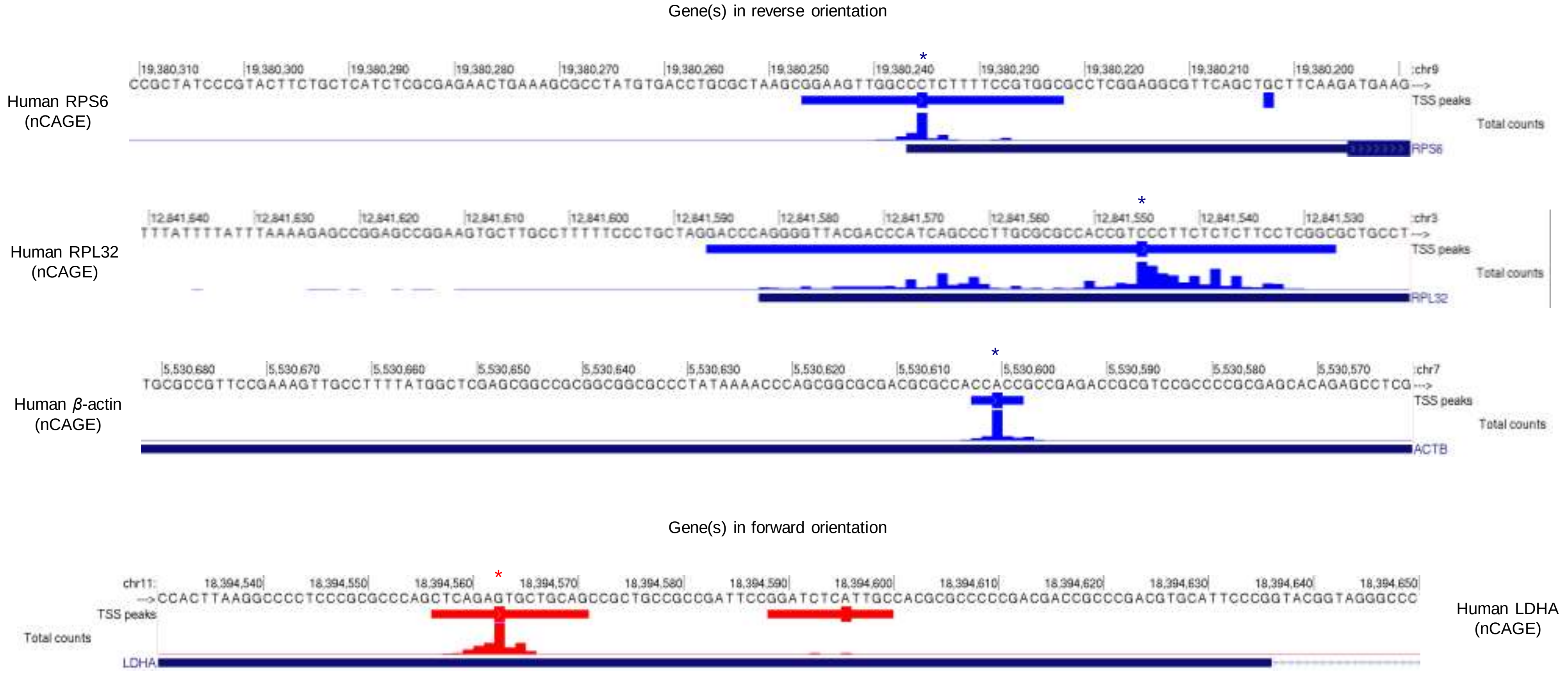

B

Gene	Dominant TSS 5'UTR START
Human RPS6	5'**C**TCTTTTCCGTGGCGCCTCGGAGGCGTTCAGCTGCTTCAAGATG-3'
Human RPL32	5'**CC**CTTCTCTCTTGGCGCTGCCTACGGAGGTGGCAGCCATCTCCTTCTCGGCATCATG-3'
Human β-actin	5'**A**CCGCCGAGACCGCGUCCGCCCCGCGAGCACAGAGCCTCGCCTTTGCCGATCCGCCGCCCGTCCACACCCGCCGCCAGCTCACCATG-3'
Human LDHA	5'**G**TCTGCCGGTCGGTTGTCTGGCTGCGCGCGCCACCCGGGCCTCTCCAGTGCCCCGCCTGGCTCGGCATCCACCCCCAGCCCGACTCACACGTGGGTTCCCGCACGTCCGCCGGCCCCCCCCGCTGACGTCAGCATAG CTGTtCCACTTAAGGCCCCTCCCGCGCCCAGCTCAGGTGCTGCAGCCGCTGCCGCCGATTCCGGATCTCATTGCCACGCGCCCCCGACGACCGCCCGACGTGCATTCCCGATTCCTTTTGGTTCCAAGTCCAATATG-3'

Suppl. Figure 2. CAGE analysis of TOP mRNAs and non-TOP mRNAs.

```
CLUSTAL O(1.2.4) multiple sequence alignment  of human LARP1 and human LARP2

Human LARP1      MLWRVLLSKRPPFPHPELDFQEAPIPSCPGRLPGRKNSVALAAAPRKEPTGDREKPLPFP      60
Human LARP2      ------------------------------------------------------------      0


Human LARP1      VLAPFSNPEHSAPAKVVRAAVPKQRKGSKVGDFGDAINWPTPGEIAHKSVQPQSHKPQPT      120
Human LARP2      -----------------------------------MENWPTPSELVNTGFQSVLSQGNKK      25
                                                    *****.*:.:...*    : : .

Human LARP1      RKLPPKKDMKEQEKGEGSDSKESPKTKSDESGEEKNGDEDCQRGGQKKKGNKHKWVPLQI      180
Human LARP2      PQN-RKEKEEKVEKRSNSDSKENRETKLNGPGEN-VSEDEAQSSNQRKRANKHKWVPLHL      83
                 :   *:. :: ** ..*****. :** :  **:  .:::.* ..*:*:.********::

Human LARP1      DMKPEVPREKLASRPTRPPEPRHIPANRGEIKGSESATYVPVAPPTPAWQPEIKPEPAWH      240
Human LARP2      DVVRSESQERPGSRNSSRCQPEANKP-----------THNNRRNDTRSWKRDRE---KRD      129
                 *:  .  :*: .** :   :*.                *:      * :*: : :     .

Human LARP1      DQDETSSVKSDGAGGARASFRGRGRGRGRGRGRGRGGTRTHFDYQFGYRKFDGV-EGPRT      299
Human LARP2      DQDDVSSVRSE-GGNIRGSFRGRGRGRGRGRGRGNPRLNFDYSYGYQEHGERTDQPFQ      188
                 ***:.***:*: .*. *.*****************. * :***.:**::..   : *

Human LARP1      PKYMNNITYYFDNVSSTELYSVDQELLKDYIKRQIEYYFSVDNLERDFFLRRKMDADGFL      359
Human LARP2      TELNTSMMYYYDDGTGVQVYPVEEALLKEYIKRQIEYYFSVENLERDFFLRGKMDEQGFL      248
                 :  ..: **:*: :..::* *:: ***:***********:********* *** :***

Human LARP1      PITLIASFHRVQALTTDISLIFAALKDSKVVEIVDEKVRRREEPEKWPLPP--IVDYSQT      417
Human LARP2      PISLIAGFQRVQALTTNLNLILEALKDSTEVEIVDEKMRKKIEPEKWPIPGPPPRSVPPT      308
                 **:***.*:*******::.**: *****. *******:*:: ******:*     .   *

Human LARP1      DFSQLLNCPEFVPRQHYQKETESAPGSPRAVTPVPT-KTEEVSNLKTLPKGLSASLPDLD      476
Human LARP2      DFSQLIDCPEFVPGQAFCSHTESAPNSPRIGSPLSPKKNSETSILQAMSRGLSTSLPDLD      368
                 *****::****** * : ..*****.***  :*:   *..*.* *::: :***:******

Human LARP1      SENWIEVKKRPRPSPARPKKSEESRFSHLTSLPQQLPSQQLMSKDQDEQEELDFLFDEEM      536
Human LARP2      SEPWIEVKKRHQPAPVKLRESVSV--------PE-GSLNQLCSSEEPEQEELDFLFDEEI      419
                 ** ******* :*:*.: ::* .         *:    :** *.:: ************:

Human LARP1      EQMDGRKNTFTAWSDEESDYEIDDRDVNKILIVTQTPHYMRRHPGGDRTGNHTSRAKMSA      596
Human LARP2      EQI-GRKNTFTDWSDNDSDYEIDDQDLNKILIVTQTPPYVKKHPGGDRTGTHMSRAKITS      478
                 **: ******* ***::*******:*:********** *:::********.* ****:::

Human LARP1      ELAKVINDGLFYYEQDLWAEKFEPEYSQIKQEVENFKKVNMISREQFDTLTPEPPVDPNQ      656
Human LARP2      ELAKVINDGLYYYEQDLWMEEDENKHTAIKQEVENFKKLNLISKEQFENLTPELPFEPNQ      538
                 *********:******* *: * ::: *********:*:**:***:.**** *.:***

Human LARP1      EVPPGPPRFQQV------------PTDALANKLFGAPE--PSTIARSLPTTVPESPNYRN      702
Human LARP2      EVPVAPSQSRQGGVQGVLHIPKKDLTDELAQKLFDVSEITSAAMVHSLPTAVPESPRIHP      598
                 *** .* : :*              ** **:***.. *   :::.:****:*****. :

Human LARP1      TRTPRTPRTPQLKDSSQTSRFYPVVKEGRTLDAKMPRKRKTRHSSNPPLESHVGWVMDSR      762
Human LARP2      TRTPKTPRTPRLQDPNKTPRFYPVVKEPKAIDVKSPRKRKTRHSTNPPLECHVGWVMDSR      658
                 ****:*****:*:* .:* ******** :::*.* *********:*****.*********

Human LARP1      EHRPRTASIS---SSPSEGTPTVGSYGCTPQSLPKFQHPSHELLKENGFTQHVYHKYRRR      819
Human LARP2      DRGPGTSSVSTSNASPSEGAPLAGSYGCTPHSFPKFQHPSHELLKENGFTQQVYHKYRRR      718
                 :: * *:*:*   :*****:* .*******:*:*****************:********

Human LARP1      CLNERKRLGIGQSQEMNTLFRFWSFFLRDHFNKKMYEEFKQLALEDAKEGYRYGLECLFR      879
Human LARP2      CLSERKRLGIGQSQEMNTLFRFWSFFLRDHFNKKMYEEFRQLAWEDAKENYRYGLECLFR      778
                 **.********************************:*** *****.**********

Human LARP1      YYSYGLEKKFRLDIFKDFQEETVKDYEAGQLYGLEKFWAFLKYSKAKNLDIDPKLQEYLG      939
Human LARP2      FYSYGLEKKFRREIFQDFQEETKKDYESGQLYGLEKFWAYLKYSQSKTQSIDPKLQEYLC      838
                 :********** :**:****** ****:***********:****::*. .*********

Human LARP1      KFRRLEDFRVDPPMGEEGNHKRHSVVAGGGGGEGRKRCPSQSSSRPAAMISQPPTPPTGQ      999
Human LARP2      SFKRLEDFRVDPPISDEFGRKRHSSTSGEES--NRHRLPPNSSTKPPNAAKPTSTSELQV      896
                 .*:*********:.:* .:**** .:*  .  .*:* * :**::*    .   *

Human LARP1      PVREDAKWTSQHSNTQTLGK     1019
Human LARP2      PINSPRRNISPESSDNSH--     914
                 *:..  :  * .*. ::
```

Suppl. Figure 3. Alignment of human LARP1 and human LARP2 proteins.

Suppl. Figure 4

CLUSTAL O(1.2.4) Human LARP family amino acid sequence alignment

```
Human LARP1        MLWRVLLSKRPPFPHPELDFQEAPIPSCPGRLPGRKNSVALAAAPRKEPTGDREKPLPFP
Human LARP2        ------------------------------------------------------------
Human LARP2ΔCT     ------------------------------------------------------------
Human La           ------------------------------------------------------------
Human LARP4        ------------------------------------------------------------
Human LARP5        ------------------------------------------------------------
Human LARP6        ------------------------------------------------------------
Human LARP7        ------------------------------------------------------------


Human LARP1        VLAPFSNPEHSAPAKVVRAAVPKQRKGSKVGDFGDAINWPTPGEIAHKSVQPQSHKPQPT
Human LARP2        -----------------------------------MENWPTPSELVNTGFQSVLSQGNKK
Human LARP2ΔCT     -----------------------------------MENWPTPSELVNTGFQSVLSQGNKK
Human La           ------------------------------------------------------------
Human LARP4        ------------------------------------------------------------
Human LARP5        ------------------------------------------------------------
Human LARP6        ------------------------------------------------------------
Human LARP7        ------------------------------------------------------------


Human LARP1        RKLPPKKDMKEQEKGEGSDSKESPKTKSDESGEEKNGDEDCQRGGQKKKGNKHKWVPLQI
Human LARP2        PQN-RKEKEEKVEKRSNSDSKENRETKLNGPGEN-VSEDEAQSSNQRKRANKHKWVPLHL
Human LARP2ΔCT     PQN-RKEKEEKVEKRSNSDSKENRETKLNGPGEN-VSEDEAQSSNQRKRANKHKWVPLHL
Human La           ------------------------------------------------------------
Human LARP4        ------------------------------------------------------------
Human LARP5        -------------MTSDQDAKV-------------VAEPQTQRVQEGKDSAHLMNGPISQ
Human LARP6        ---------------------------------------MAQSGGE--------------
Human LARP7        ------------------------------------------------------------


Human LARP1        DMKPEVPREKLASRPTRPPEPRHIPANRGEIKGSESATYVPVAPPTPAWQPEI-------
Human LARP2        DVVRSESQERPGSRNSSRCQPEANKP-----------THNNRRNDTRSWKRDR-------
Human LARP2ΔCT     DVVRSESQERPGSRNSSRCQPEANKP-----------THNNRRNDTRSWKRDR-------
Human La           ------------------------------------------------------------
Human LARP4        -----------------MLLFVEQVA----------SKGTGLNPNAKVWQEIAPGNTDAT
Human LARP5        ----------TTSQTSSIPPLSQVPA----------TKVSELNPNAEVWGAPVLH-LEAS
Human LARP6        ----------------ARPGPKT------------------------AVQ----------
Human LARP7        ------------------------------------------------------------


Human LARP1        ----KPEPAWHDQDETSSVKSDGAGGARASFRGRGRGRGRGRGRGRGGTRTHFDYQFGYR
Human LARP2        ----E---KRDDQDDVSSVRSE-GGNIRGSFRGRGRGRGRGRGRGRGNPRLNFDYSYGYQ
Human LARP2ΔCT     ----E---KRDDQDDVSSVRSE-GGNIRGSFRGRGRGRGRGRGRGRGNPRLNFDYSYGYQ
Human La           ------------------------------------------------------------
Human LARP4        PVTHGTESSWHEIAATSGAHP-E-G---------------NAELSE-DICKE--YEVMYS
Human LARP5        SAADGVSAAWEEVAGHHADRGPQ-G---------------SDANGDGDQGHE--NAALP-
Human LARP6        -----IRVAIQEAEDVDELEDEEEG---------------AETRGAGDPARYLSPGWGSA
Human LARP7        ------------------------------------------------------------

Human LARP1        KFD--------------GV-EGPRTPKYMNNITYYFDNVSSTELYSVDQELLKDYIKRQI
Human LARP2        EHG--------------ERTDQPFQTELNTSMMYYYDDGTGVQVYPVEEALLKEYIKRQI
Human LARP2ΔCT     EHG--------------ERTDQPFQTELNTSMMYYYDDGTGVQVYPVEEALLKEYIKRQI
Human La           --------------------------------------MAE-NGDNEKMAALEAKICHQI
Human LARP4        SSCETTRNTTGIEESTDGMILGPEDLS---YQ--IYD-VSGESNSAVSTEDLKECLKKQL
Human LARP5        D------PQESDPADMNALALGPSEYD---SLPENSE-TGGNESQPDSQEDPREVLKKTL
Human LARP6        SEE--------------EPSRGHSG-TT-ASGGENEREDLE-QEWKPPDEELIKKLVDQI
Human LARP7        --M--------------ETESGNQE-KVMEEESTEKKKEVE-KKKRSRVKQVLADIAKQV
                                                           .            :   :

Human LARP1        EYYFSVDNLERDFFLRRKMD--ADGFLPITLIASFHRVQALTTDISLIFAALKDSKVVEI
Human LARP2        EYYFSVENLERDFFLRGKMD--EQGFLPISLIAGFQRVQALTTNLNLILEALKDSTEVEI
Human LARP2ΔCT     EYYFSVENLERDFFLRGKMD--EQGFLPISLIAGFQRVQALTTNLNLILEALKDSTEVEI
Human La           EYYFGDFNLPRDKFLKEQIK-LDEGWVPLEIMIKFNRLNRLTTDFNVIVEALSKSKAELM
Human LARP4        EFCFSRENLSKDLYLISQMD--SDQFIPIWTVANMEEIKKLTTDPDLILEVLRSSPMVQV
Human LARP5        EFCLSRENLASDMYLISQMD--SDQYVPITTVANLDHIKKLSTDVDLIVEVLRSLPLVQV
Human LARP6        EFYFSDENLEKDAFLLKHVRRNKLGYVSVKLLTSFKKVKHLTRDWRTTAHALKYSVVL—
Human LARP7        DFWFGDANLHKDRFLREQIEKSRDGYVDISLLVSFNKMKKLTTDGKLIARALRSSAVVEL
                   :: :.  **  * :*  ::      :: :  :  :..:: *: :      .*

Human LARP1        --VDEKVRRREE---------PEKWPLPP--IVDYSQTDFSQ------------------
Human LARP2        --VDEKMRKKIE---------PEKWPIPGPPPRSVPPTDFSQ------------------
Human LARP2ΔCT     --VDEKMRKKIE---------PEKWPIPGPPPRSVPPTDFSQ------------------
Human La           EISEDKTKIRRS---------PSK-PLP--EVTDEYKNDVKNRSVYIK------GFP---
Human LARP4        DEKGEKVRPSHKRCIVILREIPETTPIE--EVKGLFKSENCP------------------
Human LARP5        DEKGEKVRPNQNRCIVILREISESTPVE--EVEALFKGDNLP------------------
Human LARP6        ELNEDHRKVRRT---------TPV-PLF--PNENLPSKMLLVYDLYLSPKLWALATPQKN
Human LARP7        DL--EGTRIRRK-----------K-PLG--E----RPKDEDERTVYVE------LLP---
                       :  :                 *:

Human LARP1        --------------LLNCPEFVPRQHYQKETESAPGSPRAVTPVPT-KTEEVSNLKTLPK
Human LARP2        --------------LIDCPEFVPGQAFCSHTESAPNSPRIGSPLSPKKNSETSILQAMSR
Human LARP2ΔCT     --------------LIDCPEFVPGQAFCSHTGNIFSSRANDVTVGYLVQ--FTYLFIYF-
Human La           ---TDATLDDIKEWLEDKGQVLNIQM-----------RRT---L-HKAFKGSIFVVFDSI
Human LARP4a       -KVISCEFAHNSNWYITFQ---------------------------------------SD
Human LARP4b       -KFINCEFAYNDNWFITFE---------------------------------------TE
Human LARP6        GRVQEKVMEHLLKLFGTFGVISSVRILKPGRELPPDIRRISSRYSQVGTQECAIVEFEEV
Human LARP7        ---KNVNHSWIERVFGKCGNVVYISI-----------PHY---KSTGDPKGFAFVEFETK


Human LARP1        GLSAS------LPDLDSE--N-WIEVKKRPRPSPARPKKSEESRFSHLTSLPQQLPSQQL
Human LARP2        GLSTS------LPDLDSE--P-WIEVKKRHQPAPVKLRESVSV--------PE-GSLNQL
Human LARP2ΔCT     --------------------Q-FI------------------------------------
Human La           ESAKK---FVETPGQKYKE-TDLLIL-FKDDYF-A--KKNEERKQNKV------------
Human LARP4        TDAQQAFKYLREEVK-----------TFQGKPIMARIK----A---INTFFAKNGY-RLM
Human LARP5        ADAQQAYKYLREEVK-----------TFQGKPIKARIKAKAIA---INTFLPKNGF-RPL
Human LARP6        EAAIKAHEFMITESQGKENMKAVLIGMKPPKKKPAKDKNHDEEP----T-----AS-IHL
Human LARP7        EQAAKAIEFLNNPPEEAPRKPGIFPKTVKNKPIPALRVVEEKKKKKKKK-----GR-MKK
```

```
Human LARP1      MSKDQDE----QEELDFLFDEEMEQMDGRKN--------TFTAWSDEESDYEI-------
Human LARP2      CSSEEPE----QEELDFLFDEEIEQI-GRKN--------TFTDWSDNDSDYEI-------
Human LARP2ΔCT   ------------------------------------------------------------
Human La         EAKLRAK----QEQEA-------------KQ--------KLE----EDAEMKS-------
Human LARP4      DSSIYSHPIQTQAQYASPV--FMQPVYNPHQQYSVYSIV-PQSWSPNPTPYFETPLAPFP
Human LARP5      DVSLYA-----QQRYATSF--YFPPMYSPQQQFPLYSLITPQTWSATHSYLDPPLVTPFP
Human LARP6      NKSLNKR----VEELQ--------YM-GD-----------------ESSANSS-------
Human LARP7      EDNIQAK----EENMDTS-NTSISKM-KRSR--------PTS----EGSDIES-------


Human LARP1      ----------------DDRDVNKILIVTQTP-------H----YMRRHPGGDR-------
Human LARP2      ----------------DDQDLNKILIVTQTP-------P----YVKKHPGGDR-------
Human LARP2ΔCT   ------------------------------------------------------------
Human La         ----------------LEEKIGCLLKFSG--------------DLDDQTCREDLHILFSN
Human LARP4      NGSFVNGFNSPGSYKTNAAAMNMGRPFQK---N------RVKPQFR-SSGGSE-HSTEGS
Human LARP5      NTGFINGFTSPAFKPAASP-LTSLRQYPPRSRN------PSKSHLR-HA--------IPS
Human LARP6      ----------------SDPESNPTSPMAGRRHAATNKLS---------PSGHQNLFLSPN
Human LARP7      ----------------TEPQKQCSKKKKKRDRVEASSLPEVRTGKRKRSSSEDAESLAPR


Human LARP1      ---------------------TGNHT--SRAKMSAELAKVINDGLFYY--EQDLWAEK-F
Human LARP2      ---------------------TGTHM--SRAKITSELAKVINDGLYYY--EQDLWMEE-D
Human LARP2ΔCT   ------------------------------------------------------------
Human La         H----GEIKW---IDFVRGAKEGIILFKEKAKEALGKAKDANNGNLQLRNKEVTWEVLEG
Human LARP4      VSLGDGQLNRYSSRNFPAERHNPTVT-GHQ-------------EQTYLQKETS-----TL
Human LARP5      AERGPGLLESPSIFNFTADRLINGVR-SPQTRQAGQTRTRIQNPSAYAKREAGPGRVEPG
Human LARP6      AS--PCTSPW---SSPLAQR-----------KGVSRKSPLAEEGRLNCSTSPEIFRKCMD
Human LARP7      -------------------SKVKKIIQKDIIKEASEASKE--NRDIEISTEE---EKDTG


Human LARP1      EPEYSQIKQEVENFKK--------------VNMISREQFDTLTPEPPVDPNQEVPPGPPR
Human LARP2      ENKHTAIKQEVENFKK--------------LNLISKEQFENLTPELPFEPNQEVPVAPSQ
Human LARP2ΔCT   ------------------------------------------------------------
Human La         EVEKEALK------------------KIIEDQQESLNKWKSKGRRFK--GKG---KGNKA
Human LARP4      QVEQNGDY--GRGRRTLFRGRRRREDDRISRPHPSTAESKAPTPKFDLLA-SNFPPLPGS
Human LARP5      SLESSPGL--GRGRKNSFGYRKKREEKFTSSQTQSPTPPKPPSPSFELGL-SSFPPLPGA
Human LARP6      YSSDSS-------------------------VTPSGSPWVRRRRQA--------------
Human LARP7      DLKDSSLLKTKRKHKKKHKERHKMGEEVIPLRVLSKSEWMDLKKEYL--ALQ---KASMA

Human LARP1      FQQV------------PTDALANKLFGAPE---PSTIARSLPTTVPESPNYRNTRTPR-T
Human LARP2      SRQGGVQGVLHIPKKDLTDELAQKLFDVSEI-TSAAMVHSLPTAVPESPRIHPTRTPK-T
Human LARP2ΔCT   ------------------------------------------------------------
Human La         AQPGSGKGKVQFQGKK------TKFASDDE------HDEHDENGATGPVKRAREETDKEE
Human LARP4      SS--RMPGELVLEN-----RMSDVVKGVYKEKDNEEL--TISCPVPADEQTECTSAQQLN
Human LARP5      AG--NLKTEDLFEN-----RLSSLIIGPSKERTLSAD--ASVNTLPVVVSR--------E
Human LARP6      ------------------------------------------------------------
Human LARP7      SL----------KKTI------SQIKSESE------M—ETDSGVPQNTGMKNEKTANRE

Human LARP1      P----RTP-QLKDSSQTSRFYPVVKEGRTLDAKMPRKRKTRHSSNPPLESHVGWVMDSRE
Human LARP2      P----RTP-RLQDPNKTPRFYPVVKEPKAIDVKSPRKRKTRHSTNPPLECHVGWVMDSRD
Human LARP2ΔCT   ------------------------------------------------------------
Human La         P----ASKQQKTENGAGDQ-----------------------------------------
Human LARP4      MSTSSPCAAELTALSTTQQEKDLIEDSS-----VQKDGLNQTT--------IPVSPPSTT
Human LARP5      PSVPASCAVSATYE-RSPSPAHLPDDPK-----VAEKQRETHS---------------VD
Human LARP6      -----EMGTQEKSPGTSPLLSRKMQTADGL----------------------PVGVLRLP
Human LARP7      -----ECRTQEKVNATGPQFV------SGV----------------------IVKIISTE

Human LARP1      HRPRTASIS---SSPS---EG---TPTVGSYGCTPQSLPKFQHPSHELLKENGFTQHVYH
Human LARP2      RGPGTSSVSTSNASPS---EG---APLAGSYGCTPHSFPKFQHPSHELLKENGFTQQVYH
Human LARP2ΔCT   ------------------------------------------------------------
Human La         ------------------------------------------------------------
Human LARP4      KPSRASTASPCNNNINAATAVALQEPRKLSYAEVCQ--KPPKEPSSVLV----QPLRELR
Human LARP5      RLPSALTATACKSVQ---VNGAATELRKPSYAEICQ--RTSKEPPSSPL----QPQKEQK
Human LARP6      RGPD----------------------------NT----RGFHGHERSRA-----------
Human LARP7      PLPGRKQVRDTLAAIS---EVLYVDLLEGDTECH----ARFKTPEDAQAVINAYTE----

Human LARP1      KYRRRCLNERKRLGIGQSQEMNTLFRFWSFFLRDHFNKKMYEEFKQLALE---DAKEGYR
Human LARP2      KYRRRCLSERKRLGIGQSQEMNTLFRFWSFFLRDHFNKKMYEEFRQLAWE---DAKENYR
Human LARP2ΔCT   ------------------------------------------------------------
Human La         ------------------------------------------------------------
Human LARP4      SNVVSPTKNEDNGAPENSVEK---------------------------------------
Human LARP5      PNTVGCGKEEKKL------AE---------------------------------------
Human LARP6      -----CV-----------------------------------------------------
Human LARP7      INKKHCWKLEIL-------SGDHEQRYWQKILVDRQAKLNQPREKKRGTEKLITKAEKIR


Human LARP1      Y----GLECLFRYYSYGLEKKFRLDIFKDFQEETVKDYEAGQLYGLEKFWAFLKYSKAKN
Human LARP2      Y----GLECLFRFYSYGLEKKFRREIFQDFQEETKKDYESGQLYGLEKFWAYLKYSQSKT
Human LARP2ΔCT   ------------------------------------------------------------
Human La         ------------------------------------------------------------
Human LARP4      ------------------------------------------------------------
Human LARP5      ------------------------------------------------------------
Human LARP6      ------------------------------------------------------------
Human LARP7      LAKTQQASKHIRFSEYD-------------------------------------------


Human LARP1      LDIDPKLQEYLGKFRRLEDFRVDPPMGEEGNHKRHSVVAGGGGGEGRKRCPSQSSSRPAA
Human LARP2      QSIDPKLQEYLCSFKRLEDFRVDPPISDEFGRKRHSSTSGEES--NRHRLPPNSSTKPPN
Human LARP2ΔCT   ------------------------------------------------------------
Human La         ------------------------------------------------------------
Human LARP4      ----PHEKPEARASKDYSGFRGNIIPRGAAGKIREQ------------RRQFSHRAIPQG
Human LARP5      ----PA-----------ERYREPPALKSTPGAPRDQ------------RRPAGGRPSPSA
Human LARP6      ------------------------------------------------------------
Human LARP7      ------------------------------------------------------------


Human LARP1      MISQPPTPPTGQPVREDAKWTSQHSNTQTLGK
Human LARP2      AAKPTSTSELQVPINSPRRNISPESSDNSH—
Human LARP2ΔCT   --------------------------------
Human La         --------------------------------
Human LARP4      VTRR-NGKEQYVPPRSPK--------------
Human LARP5      MGKR-LSREQSTPPKSPQ--------------
Human LARP6      --------------------------------
Human LARP7      --------------------------------
```

Suppl. Figure 4. Amino acid sequence alignment of human LARP superfamily protein members.

Clustal W alignment of LARP1 across eukaryotes

```
M.musculus          MATQVEPLLPAGAPLLQAEEHGLARKKPAPDAQAESGPGDGGGEPDGGVRRPRPACARPG
H.sapiens           ------------------------------------------MLWRVLLSKRPPFPHPE
G.gallus            ------------------------------------------------------------
X.tropicalis        ---------------------------------MAAQVEAPLLPANPLLPPLQQQPPPE
D.rerio             ----------------------------------------MATEVETLPPNNPLLQPEE


M.musculus          RDGAERESPRPPAAAEAPAGSDGEDGGRRDFVEAPPPKVNPWTKHAPPPAAVNGQPPPEP
H.sapiens           LDFQEAPIPSCPGRLPGRKNSVALAAAPRKEPTGDREKPLPFPVLAP-------FSNPEH
G.gallus            ------------------------------------------------------------
X.tropicalis        EMIEERRPEGGKQRQAKEDGGTGGEEVQLRKEFVEAPPPKVNPWMKAGGSTVNGQLPAEP
D.rerio             PQHKDGEYPLQERHTADSGSPGAGQEEQAAGREISEAPLPKVNPWTKKMSGVNGQPAAEH


M.musculus          SAPAKVVRAAAPKPRKGSKVGDFGDAVNWPTPGEIAHKSVQPQSHKPQPARKLPPKKDMK
H.sapiens           SAPAKVVRAAVPKQRKGSKVGDFGDAINWPTPGEIAHKSVQPQSHKPQPTRKLPPKKDMK
G.gallus            ----------------------------------------------------------MK
X.tropicalis        PVPAKVVKAGNSRPRRGSKVGDFGDATNWPTPGEIAHKTVQPPTKLQPGR-----KSLGK
D.rerio             SGPTKVVKAGNSRLRRGGKVGDFGDTNNWPTPGEIATKEVQVLKKP---------TVKRE
                                                                           :

                                   Cluster I
                                    148 151
M.musculus          EQEKGDGSDSKESPKTKSDESGEEKNGDEDCQRGGQKKKGSKHKWVPLQIDMKPEVPREK
H.sapiens           EQEKGEGSDSKESPKTKSDESGEEKNGDEDCQRGGQKKKGNKHKWVPLQIDMKPEVPREK
G.gallus            EQEKGDGSDGKESLKTKSDESGEEKNGDDDNQKSAQKKKGNKHKWVPLQIDMKSEVPRDK
X.tropicalis        KDMKEESADGKENQRVKSDESSEEKNGDDDGQRSANKKKGNKHKWVPLQIEMKPDGPREK
D.rerio             PKGKRESEESKENLKSKSDDSGEDKNTDEESQRSSHRRRGNKQRWVPLMIEVKAEGPRER
                     . * :. :.**. : ***:*.*:** *:: *:..::::*.*::**** *::*.: **::

                                                                           Cluster
                                                                              247
M.musculus          LASRPTRPQE-PRHTPAVRGEMKGSEPATYMPVSVAPPTPAWQPETKVEPAWHDQDETSS
H.sapiens           LASRPTRPPE-PRHIPANRGEIKGSESATYVPVAPP--TPAWQPEIKPEPAWHDQDETSS
G.gallus            TASRNNRQNEQHRHLPNNRGELKG-----------------WHPDNKHERPWLDHDETSS
X.tropicalis        NASRNNRQNEQQRHPLNRSD------------------PKTMYPDNKSERDWQDFDDTSS
D.rerio             SASRNNTRPYDAHRGTHSSRNGLR----------------DWPSERFEQELKDDHDEVSS
                     *** .      ::                             .:   :    * *:.**

                     II
                     250
M.musculus          VKSDGAGGARASFRGRGRGRGRGRGRGGTRTHFDYQFGYRKFDGTEGPRTHKYMNNIT
H.sapiens           VKSDGAGGARASFRGRGRGRGRGRGRGGTRTHFDYQFGYRKFDGVEGPRTPKYMNNIT
G.gallus            VKSEGG-AVRGAFRGRGRGRGRGRG---GHFDYQYGYRKFEGSDGSRTQKYASNIT
X.tropicalis        VKSEGA--IRGGFRGRGRGRGRGRGRGGTRSHFDYPYGYRKFDGSDVTRGPKFLSNIT
D.rerio             VKSDGAPYRAG---ARGRGRGRGRGRGRGHYDYYS-YKGSEGKDGAYAQKFGNSMT
                    **  .     .   .*************     *:**   *:  :* : .   *: ..:*


M.musculus          YYFDNVSSNEIYSMDQELLKDYIKRQIEYYFSVDNLERDFFLRRKMDADGFLPITLIASF
H.sapiens           YYFDNVSSTELYSVDQELLKDYIKRQIEYYFSVDNLERDFFLRRKMDADGFLPITLIASF
G.gallus            YYFDNISSTELYSVDQELLKDYIKRQIEYYFSVDNLERDFFLRRKMDSDGFLPITLIASF
X.tropicalis        YYYDNMSSSELYSVDQELLKDYIKRQIEYYFSVDNLQRDFFLRRKMDSEGFLPVGLIASF
D.rerio             YYYDNMSSAELYSVDQDLLKDYIKRQIEYYFSVDNLERDFFLRRKMDEGGFLPVSLIASF
                    **:**:** *:**:**:*******************:**********  ****: *****

                                                                        PAM2
                                                                        420-
M.musculus          HRVQALTTDISLIFAALKDSKVVEMVEEKVRRREEPEKWPLPGPPIVDYSQTDFSQLLNC
H.sapiens           HRVQALTTDISLIFAALKDSKVVEIVDEKVRRREEPEKWPLP--PIVDYSQTDFSQLLNC
G.gallus            HRVQALTTDISLIIKALKDSKVVEIVDQKIRRKEQPEKWALPGPPMADYTQTDFSQFINC
X.tropicalis        HRVQALTTDIELIVKALKDSKVVEIIDEKIRRKDQPDMWPLPGPSLSDASQTDFNQLINC
D.rerio             HRVQALTTDVSLILQALKDSKVVDIIDMKMRCKEEPQKWPLPDLCLPDQAQTDFAQFIHC
                    *********:.**. ********:::: *:* :::*: *.**   : * :**** *:::*

                     motif                  Cluster III
                     -430     438 440 444 449                   471
M.musculus          PEFVPRQHYQKETESAPGSPRAVTPVP-TKTEEVSNLKTLPKGLSASLPDLDSESWIEVK
H.sapiens           PEFVPRQHYQKETESAPGSPRAVTPVP-TKTEEVSNLKTLPKGLSASLPDLDSENWIEVK
G.gallus            PEFVPRQSFQKETESAPGSPRPTSPVP-TKTEEVSNLKTMPKGLSTSLPDLDSETWIEVK
X.tropicalis        PEFVPRQGFQKETESAPGSPRTSSPARTTKSEEVSNLKTMPKGLSASLPDLDSDNWIEVK
D.rerio             PEFIPR--HLPASPSPAGTPRSGTPRSSTPHQELCSLKMMPKGLSASLPDLDSECWIEVK
                    ***:**  .   : *..*:**. :*   *  :*:..** :*****:*******: *****


M.musculus          KRPRPSPARPKKPEEPRFSHPTALPQQLPSQQLMSKDQDEQEELDFLFDEEMEQMDGRKN
H.sapiens           KRPRPSPARPKKSEESRFSHLTSLPQQLPSQQLMSKDQDEQEELDFLFDEEMEQMDGRKN
G.gallus            KRPRPSPARPKKPEES-----------KTPQQQKQKDQDEPEELDFLFDEEMEQMDGRKN
X.tropicalis        KRPRPSPARTKDEVKS----PQVVTPLQVTPQKPPKDQDEPEELDFMFDEEMEQMDGRKN
D.rerio             KRPRPSPARPKKEDVR---------CVLQMGQVSAEEQEEPEELDFMFDEEMQQMDGRRN
                    *********.*.                   *   ::*:* *****:*****:*****:*

                       Cluster IV
                       550 554 556
M.musculus          TFTAWSEEDSDYEIDDRDVNKILIVTQTPPYMRRHPGGDRTGNHTSRAKMSAELAKVIND
H.sapiens           TFTAWSDEESDYEIDDRDVNKILIVTQTPHYMRRHPGGDRTGNHTSRAKMSAELAKVIND
G.gallus            TFTDWSDEDSDYEIDDRDVNKILIVTQTPPYLRRHPGGDRTGNHTSRAKMSSELAKVIND
X.tropicalis        TFTDWSDEDSDYEIDDRDVNKILIVTQTPPSLRKHPGGDRTGNHTSRAKMSSEFAKVIND
D.rerio             TFTDWSDEDSDCEIDDRDVNKILIVTQTPPYLRKHPGGDRTGHHTSRSKLSSELVKIIND
                    *** **:*:** *****************  :*:********:****:*:*:*:.*:***


M.musculus          GLFYYEQDLWTEKFEPEYSQIKQEVENFKKVNMISREQFDTLTPEPPVDPNQEVPPGPPR
H.sapiens           GLFYYEQDLWAEKFEPEYSQIKQEVENFKKVNMISREQFDTLTPEPPVDPNQEVPPGPPR
G.gallus            GLYYYEQDLWTEQFEPEYSQIKQEVENFKKVNMISREQFDTLTPEPPVDPNQEVPPGPPR
X.tropicalis        GLFYYEQDLWTEQFEPEYSQIKQEVENFKKVNMISREQFDTLTPEPPVDPNQEVPPGPPR
D.rerio             GLFYYEQDLWDDSGEPEYAIIKQEVENFKKVHLISREQFDCLTPEPPVDPNQEVPPGPPR
                    **:******* :. ****: ***********::******* *******************
```

```
                                               Cluster V
                                             689 692 697 700
M.musculus       FQQVPTDALANKLFGAPEPSTIARSLPTTVPESPNYRNARTPRTPRTPQLKDSSQTPRFY
H.sapiens        FQQVPTDALANKLFGAPEPSTIARSLPTTVPESPNYRNTRTPRTPRTPQLKDSSQTSRFY
G.gallus         FQQVPTDALANKLFGVPEPSTIARSLPTTVPESPNYRNARTPRTPRTPQLKDPTQTPRFY
X.tropicalis     FQQVPTDDLANKLFGAPEPSTIARSLPTTVPESPNYRNARTPRTPRTPRLKDPTLTPRFY
D.rerio          PTQIPTEALANKLFGAPDPSCMARSLPTTVPDSPSHRSARTPRTPRTPRLKD-AQTPRFY
                   *:**: *******.*:** :*********:**.:*.:*********:*** : *.***

                                                         Cluster VI
                                                      768     774
                                                      770     776
                                       747            772     779
M.musculus       PVVKEGRTLDAKMPRKRKTRHSSNPPLESHVGWVMDSREHRPRTASIS--SSPSEGTPAV
H.sapiens        PVVKEGRTLDAKMPRKRKTRHSSNPPLESHVGWVMDSREHRPRTASIS--SSPSEGTPTV
G.gallus         PVVKEGRTIDAKTPRKRKTRHSSNPPMECHVGWVMDSREHRPRTASIS--SSPSEGTPAV
X.tropicalis     PVVKEGRTIDAKTPRKRKTRHSSNPPMECHVGWVMDSREHRPRTASVSSTASPSEVAPAL
D.rerio          PVVKDGRPLDAKTPRKRKTRHSSNPPMECHVGWVMDSREHRSRTASVSSNASPSEGTAAM
                 ****:**.:*** *************:*.************.****:*  :**** :.::

                 Cluster VI
                  784  788
                  785  791
M.musculus       GSYGCTPQSLPKFQHPSHELLKENGFTQHVYHKYRRRCLNERKRLGIGQSQEMNTLFRFW
H.sapiens        GSYGCTPQSLPKFQHPSHELLKENGFTQHVYHKYRRRCLNERKRLGIGQSQEMNTLFRFW
G.gallus         GSYGCTPQSLPKFQHPSHELLKENGFTQHVYHKYRRRCLNERKRLGIGQSQEMNTLFRFW
X.tropicalis     SNYGCTPQSLPKFQHPSHELLKENGFTQHVYHKYRRRCLNERKRLGIGQSQEMNTLFRFW
D.rerio          GNFGCTPQSLPKFQHPSHELLKENGFTQHVYHKYRRRCLNERKRLGIGQSQEMNTLFRFW
                 ..:**********************************************************

M.musculus       SFFLRDHFNKKMYEEFKQLALEDAKEGYRYGLECLFRYYSYGLEKKFRLDIFKDFQEETV
H.sapiens        SFFLRDHFNKKMYEEFKQLALEDAKEGYRYGLECLFRYYSYGLEKKFRLDIFKDFQEETV
G.gallus         SFFLRDHFNKKMYEEFKQLAVEDGKEGYRYGLECLFRYYSYGLEKKFRPDIFKDFQEETI
X.tropicalis     SFFLRDHFNKKMYEEFRQLAVEDSKEGYRYGLECLFRYYSYGLEKKFRVDIFRDFQEETR
D.rerio          SFFLRDHFNRKMYEEFRQLSVEDGKEGYRYGLECLFRFYSYGLERKFRPDIFKDFQEETV
                 *********:******:**::**.*************:******:*** ***:******

M.musculus       KDYEAGQLYGLEKFWAFLKYSKAKNLDIDPKLQEYLGKFRRLEDFRVDPPMGEEGNHKRH
H.sapiens        KDYEAGQLYGLEKFWAFLKYSKAKNLDIDPKLQEYLGKFRRLEDFRVDPPMGEEGNHKRH
G.gallus         KDYEAGQLYGLEKFWAFLKYSKAKNLDINSKLQEYLSKFKRLEDFRVDPPMGEEGGHKRY
X.tropicalis     RDYETGQLYGLEKFWAFLKYSKAKNLDIDPILQEYLSKFRRLEDFRVDPPMGEDGGSKRR
D.rerio          KDYEAGQLYGLEKFWAFLKYSKTKNMEIEPKLQEHLSKFRRLEDFRVDPPMEDGVRKRHS
                 :***:*****************:**::*:.  ***:*.**:*********** :    ::

                                 Cluster VII
                            979           994
M.musculus       PVVAGGSG-EGRKRCPSQSSSRPATGISQPPTTPTG-QATREDAKWTSQHSDTLTLRK--
H.sapiens        SVVAGGGGGEGRKRCPSQSSSRPAAMISQPPTPPTG-QPVREDAKWTSQHSNTQTLGK--
G.gallus         PTTSGGDG---RKRYPSQSSSKTVSQCPSSQAHASSSQPAQEDAKNLSQQVPAVESQTVG
X.tropicalis     HSLSTGEG---RRRYPSQSSSRGSGHQTSTQNSAREEAKAQSHPSTATPDSQTPGAGK--
D.rerio          SSGDG------RRRHPSHPGSRSQTQSQLQPSGAAAGATRDDSRAQSQRDARPPK-----
                            *:* **:..*:           .       .       .  .

M.musculus       -
H.sapiens        -
G.gallus         K
X.tropicalis     -
D.rerio          -
```

Suppl. Figure 5. Conservation of rapamycin-sensitive phosphorylation sites on LARP1.
