# Supplementary material for: mTORC1 promotes TOP mRNA translation through site-specific phosphorylation of LARP1
Source: Nucleic Acids Res. 2021 Jan 4;49(6):3461–89. doi: 10.1093/nar/gkaa1239 (PMC8034618; doi:10.1093/nar/gkaa1239)
Supplement: gkaa1239_Supplemental_Files [file gkaa1239_supplemental_files.zip › 2020-10-08 Suppl. Table 1.docx]

**Suppl. Table 1.** The following oligonucleotides were employed for site-directed mutagenesis of human LARP1:

LARP1_R840E_forward

5’GGAGATGAACACACTCTTCGAATTCTGGTCCTTCTTCCTCCG-3’

LARP1_R840E_reverse

5’CGGAGGAAGAAGGACCAGAATTCGAAGAGTGTGTTCATCTCC-3’

LARP1_Y883A_forward

5’CTACAGTGCTGGCCTGGAAAAGAAGTTCCGGCTGGACATATTC-3’

LARP1_Y883A_reverse

5’CCAGGCCAGCACTGTAGTATCTATCGAAAAAGGCACTCCAAACC-3’

LARP1_S148A/S151A_forward 5’AAGGAGAGTCCAAAAACCAAAGCAGATGAAGCAGGGGAGGAAAAGAATGGAGATGAGGAT-3’

LARP1_S148A/S151A_reverse 5’ATCCTCATCTCCATTCTTTTCCTCCCCTGCTTCATCTGCTTTGGTTTTTGGACTCTCCTT-3’

LARP1_S247A/S250A_forward 5’GACCAGGATGAGACATCGGCTGTGAAGGCTGATGGGGCTGGTGGGGCGCGGGCTTCCTTC-3’

LARP1_S247A/S250A_reverse 5’GAAGGAAGCCCGCGCCCCACCAGCCCCATCAGCCTTCACAGCCGATGTCTCATCCTGGTC-3’

LARP1_T438A/S440A/S444/T449A_forward 5’GTTCCCCGTCAGCACTACCAAAAGGAGGCAGAGGCGGCACCTGGCGCTCCTCGTGCAGTCGCCCCAGTGCCAACCAAAACAGAGGAGGTC-3’

LARP1_T438A/S440A/S444/T449A_reverse

5’GACCTCCTCTGTTTTGGTTGGCACTGGGGCGACTGCACGAGGAGCGCCAGGTGCCGCCTCTGCCTCCTTTTGGTAGTGCTGACGGGGAAC-3’

LARP1_S471A_forward

5’AAGGGCCTGTCTGCCGCCCTGCCTGACCTGGAT-3’

LARP1_S471A_reverse

5’ATCCAGGTCAGGCAGGGCGGCAGACAGGCCCTT-3’

LARP1_S550A/S554A_forward 5’ACCTTCACTGCCTGGGCTGATGAGGAAGCTGACTATGAGATTGAT-3’

LARP1_S550A/S554A_reverse 5’ATCAATCTCATAGTCAGCTTCCTCATCAGCCCAGGCAGTGAAGGT-3’

LARP1_S689A/T692A/S697A_forward 5’CCCTCCACCATCGCCCGCGCTCTACCAGCCACTGTCCCAGAGGCACCAAACTACCGCGGC-3’

LARP1_S689A/T692A/S697A_reverse 5’GCCGCGGTAGTTTGGTGCCTCTGGGACAGTGGCTGGTAGAGCGCGGGCGATGGTGGAGGG-3’

LARP1_S747A_forward

5’AAGACAAGACACAGTGCAAACCCACCCTTGGAG-3’

LARP1_S747A_reverse

5’CTCCAAGGGTGGGTTTGCACTGTGTCTTGTCTT-3’

LARP1_T768A/S770A/S772A/S774A/S776A/T779A_forward 5’ATGGATTCCCGTGAGCACAGGCCCCGTGCTGCTGCCATCGCCTCCGCCCCCGCAGAAGGGGCGCCTACAGTTGGCAGCTATGGCTGTACC-3’

LARP1_T768A/S770A/S772A/S774A/S776A/T779A_reverse

5’GGTACAGCCATAGCTGCCAACTGTAGGCGCCCCTTCTGCGGGGGCGGAGGCGATGGCAGCAGCACGGGGCCTGTGCTCACGGGAATCCAT-3’

LARP1_S784A/T788A/S791A_forward

5’GGGACGCCTACAGTTGGCGCCTATGGCTGTGCCCCTCAGGCATTGCCCAAGTTCCAGCAT-3’

LARP1_S784A/T788A/S791A_reverse 5’ATGCTGGAACTTGGGCAATGCCTGAGGGGCACAGCCATAGGCGCCAACTGTAGGCGTCCC-3’

LARP1_S979A_forward

5’AGGAAGCGGTGCCCCGCCCAGTCTTCCAGCAGG-3’

LARP1_S979A_reverse

5’CCTGCTGGAAGACTGGGCGGGGCACCGCTTCCT-3’

LARP1_S689D_forward

5’CCTCCACCATCGCCCGCGATCTACCAACCACTGTCC-3’

LARP1_S689D_reverse

5’GGACAGTGGTTGGTAGATCGCGGGCGATGGTGGAGG-3’

LARP1_S697D_forward 5’CTACCAACCACTGTCCCAGAGGATCCAAACTACCGCAACACCAGG-3’

LARP1_S697D_reverse 5’CCTGGTGTTGCGGTAGTTTGGATCCTCTGGGACAGTGGTTGGTAG-3’
