# Supplementary material for: mTORC1 promotes TOP mRNA translation through site-specific phosphorylation of LARP1
Source: Nucleic Acids Res. 2021 Jan 4;49(6):3461–89. doi: 10.1093/nar/gkaa1239 (PMC8034618; doi:10.1093/nar/gkaa1239)
Supplement: gkaa1239_Supplemental_Files [file gkaa1239_supplemental_files.zip › 2020-10-08 Suppl. Table 2.docx]

**Suppl. Table 2.** The following oligonucleotides were used to sequence human LARP1 and human LARP2:

LARP1_sequencing1_forward

5’ATGCTTTGGAGGGTGCTTTTG-3’

LARP1_sequencing2_forward

5’GTTCCTAAACAGCGCAAAGGC-3’

LARP1_sequencing3_forward

5’TGCCAGCGAGGCGGGCAGAAG-3’

LARP1_sequencing4_forward

5’GACCAGGATGAGACATCGAGTG-3’)

LARP1_sequencing5_forward

5’GTGGATCAGGAACTGCTCAAAG-3’

LARP1_sequencing6_forward

5’GAGGAACCAGAAAAGTGGCCTC-3’

LARP1_sequencing7_forward

5’ATTGAAGTGAAGAAGAGGCCTC-3’

LARP1_sequencing8_forward

5’AGGGATGTCAACAAGATCCTC-3’

LARP1_sequencing9_forward

5’GAGCAGTTTGACACACTGACC-3’

LARP1_sequencing10_forward

5’TCACGGTTTTACCCAGTGGTG-3’

LARP1_sequencing11_forward

5’GAACTGCTCAAGGAAAATGGC-3’

LARP1_sequencing12_forward

5’TACAGTTATGGCCTGGAAAAG-3’

LARP1_sequencing13_forward

5’CGACACTCAGTGGTAGCAGGAG-3’

LARP1_sequencing14_reverse

5’AGGGAATGGCAATGGCTTCTC-3’
