# Supplementary material for: mTORC1 promotes TOP mRNA translation through site-specific phosphorylation of LARP1
Source: Nucleic Acids Res. 2021 Jan 4;49(6):3461–89. doi: 10.1093/nar/gkaa1239 (PMC8034618; doi:10.1093/nar/gkaa1239)
Supplement: gkaa1239_Supplemental_Files [file gkaa1239_supplemental_files.zip › 2020-10-08 Suppl. Table 3.docx]

**Suppl. Table 3.** The following oligonucleotides were used for the analysis of human RPS6, RPL32, LDHA and β-actin mRNA levels by RT-ddPCR:

RPS6_forward

5’-CTGGGTGAAGAATGGAAGGGTT-3’

RPS6_reverse

5’-TGCATCCACAATGCAACCAC-3’

RPL32_forward

5’-AGCCATCTCCTTCTCGGCAT-3’

RPL32_reverse

5’-TCAATGCCTCTGGGTTTCCG-3’

LDHA_forward

5’- AAAGGCTACACATCCTGGGC-3’

LDHA_reverse

5’-GGTGCACCCGCCTAAGATTC-3);

β-actin_forward

5’-TGATGATATCGCCGCGCTC-3’

β-actin_reverse

5’-CATCACGCCCTGGTGCC-3’
